# Supplementary material for: Reformulation of Trivers–Willard hypothesis for parental investment
Source: Commun Biol. 2022 Apr 19;5:371. doi: 10.1038/s42003-022-03286-z (PMC9018816; doi:10.1038/s42003-022-03286-z)
Supplement: Supplementary file 2 — Supplementary Materials [file 42003_2022_3286_MOESM2_ESM.pdf]

Supplementary materials to:

Reformulation of Trivers-Willard hypothesis for parental investment

Jibeom Choi[1], Hyungmin Roh[2], Sang-im Lee[3,*], Hee-Dae Kwon[4,*], Myungjoo Kang[2,5,*], Piotr G Jablonski[1,6*]

1 Evolutionary Dynamics Laboratory, Department of Applied Mathematics, Kyung Hee University, Korea
2 Samsung Research, Samsung Electronics Co., Ltd., Korea
3 Laboratory of Integrative Animal Ecology, Department of New Biology, DGIST, Korea
4 Department of Mathematics, Inha University, Korea
5 Department of Mathematical Sciences, Seoul National University, Korea
6 Laboratory of Behavioral Ecology and Evolution, School of Biological Science, Seoul National University, Korea
7 Behavioral Ecology Group, Museum and Institute of Zoology, Polish Academy of Sciences, Warsaw, Poland
* Corresponding authors

*Correspondence concerning evolutionary ecology aspects of the paper should be addressed to behavioral ecologist (PGJ: snulbee@behecolpiotrsangim.org) and animal ecologist (SIL: sangim@dgist.ac.kr); the requests and questions regarding the MATLAB code and model details should be addressed to the first author (JC: snu10@snu.ac.kr); the requests and questions regarding mathematical analysis, definitions, theorems, and proofs should be addressed to JC and to mathematicians: HDK (hdkwon@inha.ac.kr) and MK (mkang@snu.ac.kr).

Table of Contents

**Part 1**	**A brief overview of the models of TW hypothesis and assumptions**	2
**Part 2**	**Results of the computational model when fitness functions are the same among offspring**	4
**Part 3**	**Results of the computational models with two different fitness functions: *Sex 1* and *Sex 2* offspring**	
	Overview and summary of coefficients in fitness functions	6
	Detailed results of models *M3* to *M6*	8
	Detailed results of models *M7-1* to *M7-10*	13
	Visual representations of global strategy	25
	Tangent-line rule of thumb	35
**Part 4**	**Mathematical theorems and proofs**	36
	**References for Supplementary Materials**	51

Part 1: A brief overview of the previous models and assumptions

A brief overview of models of Trivers-Willard hypothesis leading to our approach

In order to theoretically understand the observed variation in empirical evidence from the tests of Trivers-Willard hypothesis (TWH), a number of theoretical models have been proposed (e.g. Carranza 2002, Cameron & Linklater 2002, Leimar 1996, Veller et al. 2016, Schindler et al. 2015, Shyu and Caswell 2018, Borgstede 2019). They used linear, convex, concave, or sigmoid 'fitness functions' describing the effect of parental condition/total available investment on the fitness of an individual offspring. They suggested that the differences in fitness functions between sexes are central for understanding the empirical cases, and that in certain conditions, reversed TW predictions are expected. However, the explicit effects of various differences in fitness functions between sexes have not been thoroughly investigated. We consider that focusing explicitly on fitness function shape may be regarded as an alternative approach to the recent population-level expansions of TWH models (Schindler et al 2015, Boergstede 2019). This approach is open to the possibility of incorporating population-level phenomena through their effects on the sex-specific fitness function shape.

Additionally, following some of the empirical literature's focus on the primary sex ratio, models up to date often focused on analyzing primary rather than secondary sex ratios, even though the secondary sex ratios are at the center of predictions of the original TWH (TWH concerns sex ratio after parental investment/at independence). Before reaching independence, the offspring are cared for/invested in according to the specific parental strategy composed of multiple elements. The full list of elements of the parental strategy contains five aspects: primary sex ratio before parental investment (*1° SR*), clutch size (the number of offspring at the beginning of the investment), rules of allocation of investment among individual offspring of both sexes, the secondary sex ratio at the end of parental investment (*2° SR*), and brood size (the number of offspring at the end of the investment). However, the theoretical models did not explore all the elements simultaneously. For example, some models did not explicitly model the number of offspring per brood or simplified the situation by assuming two offspring (Veller et al. 2016). These elements of the parental strategy are actually tightly linked. For example, investment rules can mediate differential offspring mortality, the subsequent number, and sex ratio at offspring's independence. Furthermore, optimal investment rules may depend on the primary offspring sex ratio in a brood. Finally, some combinations of primary sex ratios and the associated optimal parental investment rules yield higher parental fitness than other combinations do. This may create the natural selection for certain combinations of the primary sex ratio and the optimal allocation rules. Therefore, a thorough theoretical model that covers all those phenomena is needed, which we aim to provide here.

Assumptions of the model presented in this paper compared to previous models

To the best of our knowledge, the previously published models have not combined all the elements of parental investment strategy. Additionally, the models have not attempted to explore the interplay among these multiple elements into one comprehensive theoretical approach. To achieve this, our models have simplified reality in a manner different from the previously published models of TWH.

In contrast to some earlier models (e.g. Leimar 1996, Carranza 2002; Krist 2006), our computational models do not consider the reproduction cost which may reduce the reproductive fertility of upcoming seasons. The model therefore would be most appropriate to explain a simple semelparous organism. Hence, the fitness function has a classical logistic shape which is monotonically increasing with a certain plateau of fitness. This does not consider the parental costs and any effects (positive or negative) that the 'over-investment' may have on the fitness of an offspring. Hence, there is no trade-off built into the model that would prevent parents ('punishing' or 'enforcing' parents) from expending unnecessarily large amounts of care even though it no longer practically increases the fitness. This has to be taken into account when interpreting the figures. Any result beyond the certain investment values where all offspring are supplemented with the investment at the level of the plateau in their fitness functions should take the least attention. It is likely in reality that we would expect costs of unnecessary energy expenditure that does not bring any fitness benefits. We set the upper threshold as twice the value of the inflection point's x-value because *per capita* investment over this bound indicates an overly invested condition according to Lemma 3. This is marked with the translucent line in optimal *per capita* investment figures (panels f, i, l in Fig. S3-S16).

Unlike the model of Veller et al. (2016), we did not consider maternal effects other than investment. For example, the model does not allow to simulate the effect of maternal condition on the initial composition of brood (i.e., clutch size and primary sex ratio). Also, the model does not differentiate between multiple types of investments, which is a simplification of reality that is often implemented in other models (Rosenheim et al

1996). The model does not have any explicit population effects important in the evolution of sex ratio (e.g. Leimar 1996; Wild and West 2007; Borgstede 2019; Schindler et al 2015; Shyu and Caswell 2016); however, one can argue that these effects might be to some extent represented in the shape of the fitness functions for male and female offspring. Strictly speaking, this would require the fitness function to change shape in response to the population composition and population-level processes (which was not considered in the present study). The fitness function shape in real situations depends on multiple factors including (1) type of mating system and the future sexual attractiveness of offspring; (2) social system and the future social status of offspring; (3) sexual dimorphism and offspring growth curve shapes; (4) population-level processes such as dispersal, operational sex ratio, local mate, and local resource competition (Cockburn et al. 2002; Wild and West 2007).

In order to reduce the computational burden, *1° SR*s were set to three values (0.3, 0.5, 0.7), which has constrained the precision of predicting optimal *1° SR*s to three levels. In a situation where all values of *1° SR* are possible, the model may predict decreasing or increasing trend of optimal *1° SR* as *S* varies. This constraint could have been be resolved if much higher computation had been used. Even with the current set of models, however, we can formulate insights concerning the comprehensive multi-element parental investment strategies.

Additionally, the model does not simulate evolution and co-evolution among the elements of parental strategy. However, it allows us to conveniently visualize the possible selection pressures on each of the elements and on the combinations of them. We did not consider the fact that evolution of the postulated optimal strategies may be affected by evolutionary constraints and costs (e.g. costs of evolving the ability to manipulate the primary sex ratio; or ability to detect the sex of offspring and sex ratio of brood, etc). Finally, our model focuses on predicting optimal strategies of parents that are able to distinguish offspring sexes and are able to ‘sense’ or ‘detect’ their own condition or available investment (*S*). Obviously, it may be unrealistic to expect that animals always have such a perfect knowledge or sensory abilities. Therefore, we are aware that the theoretically optimal sex allocation rules may not always be observed in nature. Additionally, physiological constraints (Tinbergen 1963, Pen & Weissing 2002) may also prevent animals from optimally investing in offspring.

Part 2: Results of the computational model when fitness functions are the same among offspring

Results of the computational models when fitness function is the same among offspring

Here we show a full graphical account of the results from the computational models. The properties of the models are also supported by mathematical analyses (Part 4). When the fitness functions, f_{b_i}, were identical for all offspring (model *M1* and *M2*; Fig. S1a, e, Fig. 2a), we found that choosing some of the offspring from the initial number of 10 (Fig. S1c, g, Fig. 1c) and providing equitable investment (Fig. S1d, h, Fig. 2b, c) to them (hence referred to as 'selective equitable distribution' or 'SED') while abandoning the remaining offspring (x_i=0) is optimal. This was also proven mathematically (Theorem 1 and its corollary, Part 4). The results show that the number of offspring that receive the investment (equivalent to the brood size) from optimally behaving parents increases monotonically with the amount of expendable parental investment (*S*). This can be represented by the summation of step functions (Theorem 2, Part 4), and it may be relevant to the theories of the optimal clutch size (Smith & Fretwell 1974; Haig 1990). In this situation, the optimal *per capita* investment toward each offspring chosen for investment is similar to the optimal expenditure mentioned by Smith and Fretwell (1974) which is relevant to Theorem 3 (Part 4) concerning optimal *per capita* investment.

The coefficients of the fitness function in model *M1* are $= 10.6393,\ \beta = 0.25,\ \gamma = 11,\ \delta = -0.6393$ for $f(x) = \frac{\alpha}{1+e^{-\beta(x-\gamma)}} + \delta$. Those of model *M2* are $= 10.0005,\ \beta = 1,\ \gamma = 10,\ \delta = -0.0005$ for the same equation.

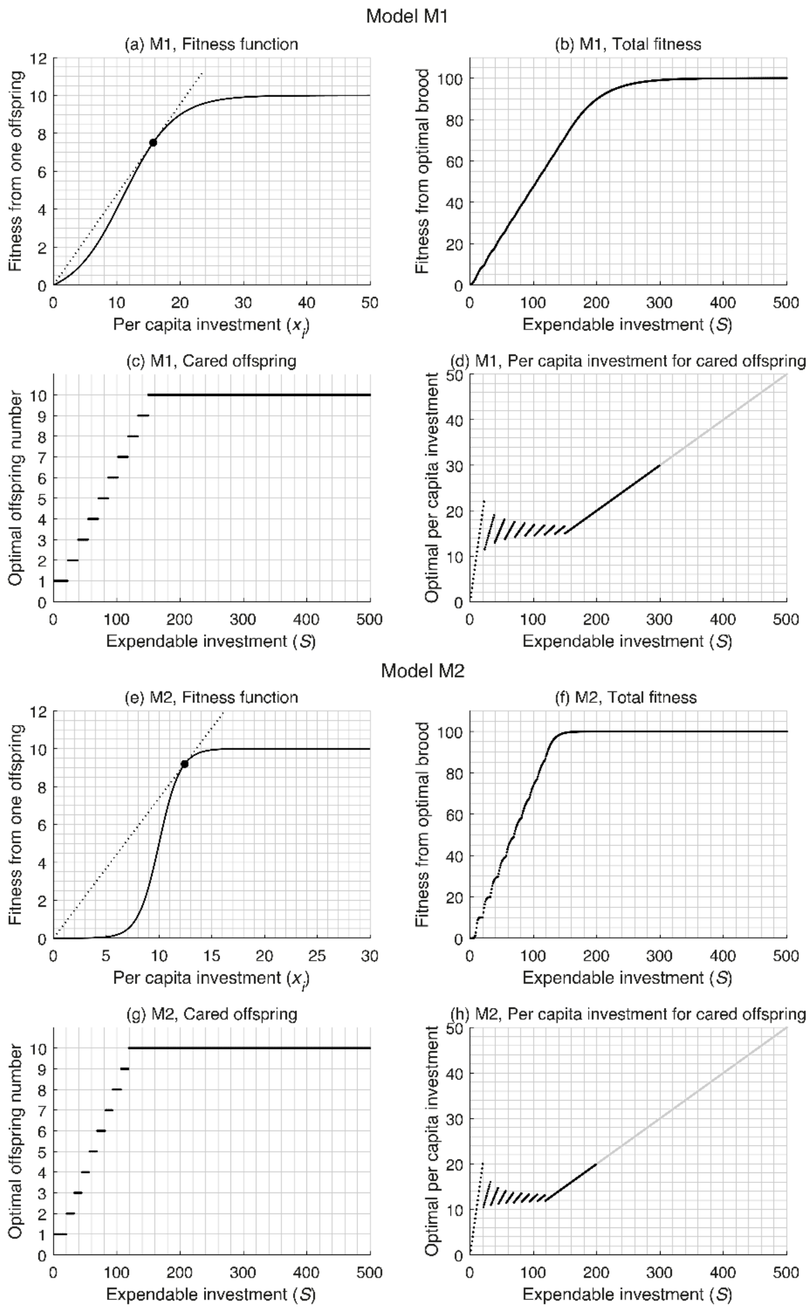

Figure S1 | Summary of models *M1* (a-d) and *M2* (e-h) with the same fitness function among all offspring. Panels (a), (b), (d) correspond to Fig. 2a, b, c in the main text.
(a) The logistic fitness function of model *M1*. In this model, every offspring has an identical fitness function. The horizontal axis represents the *per capita* investment into an offspring (x_i).
(b) The relationship between total parental investment (S) and fitness of the full brood to parents (fitness sum of all offspring in the brood) for optimally allocating parents.
(c) The relationship between total parental investment (S) and the number of offspring in a brood (offspring that are cared for) from optimally allocating parents. As expected from the mathematical theorems (Part 4), the number of offspring is expressed here as the sum of step functions.
(d) The relationship between total parental investment (S) and the *per capita* investment to each cared offspring (x_i). Approximately until S=160, the *per capita* investment stays near the most optimal point (x-value of the black circle in (a)) which is mathematically proven (Part 4). As the *per capita* investment larger than 30 was considered unrealistic considering the flatness of the fitness function, these values were marked with a translucent line.
(e) The logistic fitness function of model *M2*.
(f) The relationship between total parental investment (S) and fitness of the full brood to parents for optimally allocating parents.
(g) The relationship between total parental investment (S) and the number of offspring in a brood from optimally allocating parents. As expected from the mathematical theorems (Part 4), the number of offspring is expressed here as the sum of step functions.
(h) The relationship between total parental investment (S) and the *per capita* investment to each cared offspring (x_i). Approximately until S=120, the *per capita* investment stays near the most optimal point (x-value of the black circle in (e)) which is mathematically proven (Part 4). As the *per capita* investment larger than 20 was considered unrealistic considering the flatness of the fitness function, these values were marked with a translucent line.

Part 3: Results of the computational models with two different fitness functions: *Sex 1* and *Sex 2* offspring

Overview of the contents of Part 3

Table S1 presents coefficients of the logistic functions in the models, Fig. S2 explains the design of the figures S3-S16, where the detailed outcomes of models *M3* to *M6* and *M7-1* to *M7-10* are presented. In models *M7-1* to *M7-10*, we set the asymptote of *Sex 1* as 30 with the inflection *x*-value of 40. The asymptotes of *Sex 2* were set from 10 (*M7-1*) to 100 (*M7-10*) in a step size of 10 with the inflection *x*-value of 60. Based on the examination of these results, we made a table comparing the local optimal investment strategies (for each *1° SR*) from the predictions from the classical TWH (Table S2). Fig. S17 summarizes these analyses.

Fig. S18 explains the elements present in Fig. S19-S32. Panels (b) in Fig. S19-S32 show how the presence (solid lines) or absence (dotted lines) of optimal investment rules affects the relationship between the total available investment (*S*) and fitness from a brood for the three primary sex ratios (indicated by line color). When there is no optimization of parental allocation, all resources are equitably distributed to all offspring (outright equitable distribution). As proven computationally and mathematically, the optimal *1° SR* given outright equitable distribution follows the pattern from TWH. For mathematical analysis of the argument, see Theorem 6.

Panels (c) and (d) of Fig. S19-S32 were created by combining elements of Fig. S3-S16 in a way that helps in visualizing the 'globally' optimal strategies. Each panel (c) contains schematics of globally optimal strategy. In panel (c), line colors indicate the globally optimal *1° SR*s and values on the *y*-axis indicate the associated globally optimal *2° SR*s. The globally optimal *2° SR*s result from the ability of parents to 'choose' the combination of *1° SR* and the associated optimal parental allocation rules that globally maximize parental fitness from the brood. Each panel (d) shows how relative investments in *Sex 1* and *Sex 2* change with increasing *S* for all those combinations of *1° SR* and locally optimal allocation rules that result in globally maximized fitness accrued by parents from the brood. Horizontal bars above the panel indicate the *1° SR*s included in the globally optimal strategy. Based on the examination of Fig. S19-S32, we summarized in Table S3 the globally optimal strategies. The globally optimal strategy assumes that the parents can maximize parental fitness (panels (b) of Fig. S3-S16) by 'choosing' the optimal combination of *1° SR*s (among the three values provided in the model) and the associated locally optimal investment strategies (according to model predictions in panels (d)-(l) in Fig. S3-S16). Table S3 also compares the globally optimal investment strategies from the model to the predictions from the classical TWH.

Finally, we analyzed if there is an association between the properties of the two fitness functions and the outcome of the models (Table S4). We found out that the 'tangent-line rule of thumb' can explain some important aspects of the optimal pattern of parental investment relevant to the classical TWH. This rule can also explain empirical inconsistencies observed in the previous studies.

Table S1. Coefficients of the logistic functions and the tangent values for the tangent line (panel (a) in figures S3-S16) in the models *M3-M6* and *M7-1* to *M7-10*.

	Coefficients								The slope of tangent through the origin	
	Sex 1				*Sex 2*					
Model Name	α	β	γ	δ	α	β	γ	δ	*Sex 1*	*Sex 2*
Model *M3*	10.000 +3E-6	1.500	10.000	-3E-6	30.002	0.100	95.000	-0.002	0.794	0.231
Model *M4*	10.000 +3E-6	1.500	10.000	-3E-6	15.037	0.100	60.000	-0.037	0.794	0.165
Model *M5*	10.600	0.250	11.000	-0.600	12.030	0.500	12.000	-0.030	0.477	0.660
Model *M6*	10.200	0.200	20.000	-0.200	10.000	1.000	20.000	0.000	0.296	0.414
Model *M7-1*	30.030	0.170	40.000	-0.030	10.010	0.114	60.000	-0.010	0.512	0.114
Model *M7-2*	30.030	0.170	40.000	-0.030	20.020	0.114	60.000	-0.020	0.512	0.228
Model *M7-3*	30.030	0.170	40.000	-0.030	30.030	0.114	60.000	-0.030	0.512	0.342
Model *M7-4*	30.030	0.170	40.000	-0.030	40.040	0.114	60.000	-0.040	0.512	0.455
Model *M7-5*	30.030	0.170	40.000	-0.030	50.050	0.114	60.000	-0.050	0.512	0.569
Model *M7-6*	30.030	0.170	40.000	-0.030	60.060	0.114	60.000	-0.060	0.512	0.683
Model *M7-7*	30.030	0.170	40.000	-0.030	70.070	0.114	60.000	-0.070	0.512	0.797
Model *M7-8*	30.030	0.170	40.000	-0.030	80.080	0.114	60.000	-0.080	0.512	0.911
Model *M7-9*	30.030	0.170	40.000	-0.030	90.090	0.114	60.000	-0.090	0.512	1.025
Model *M7-10*	30.030	0.170	40.000	-0.030	100.100	0.114	60.000	-0.100	0.512	1.139

Results of all models with two sexes

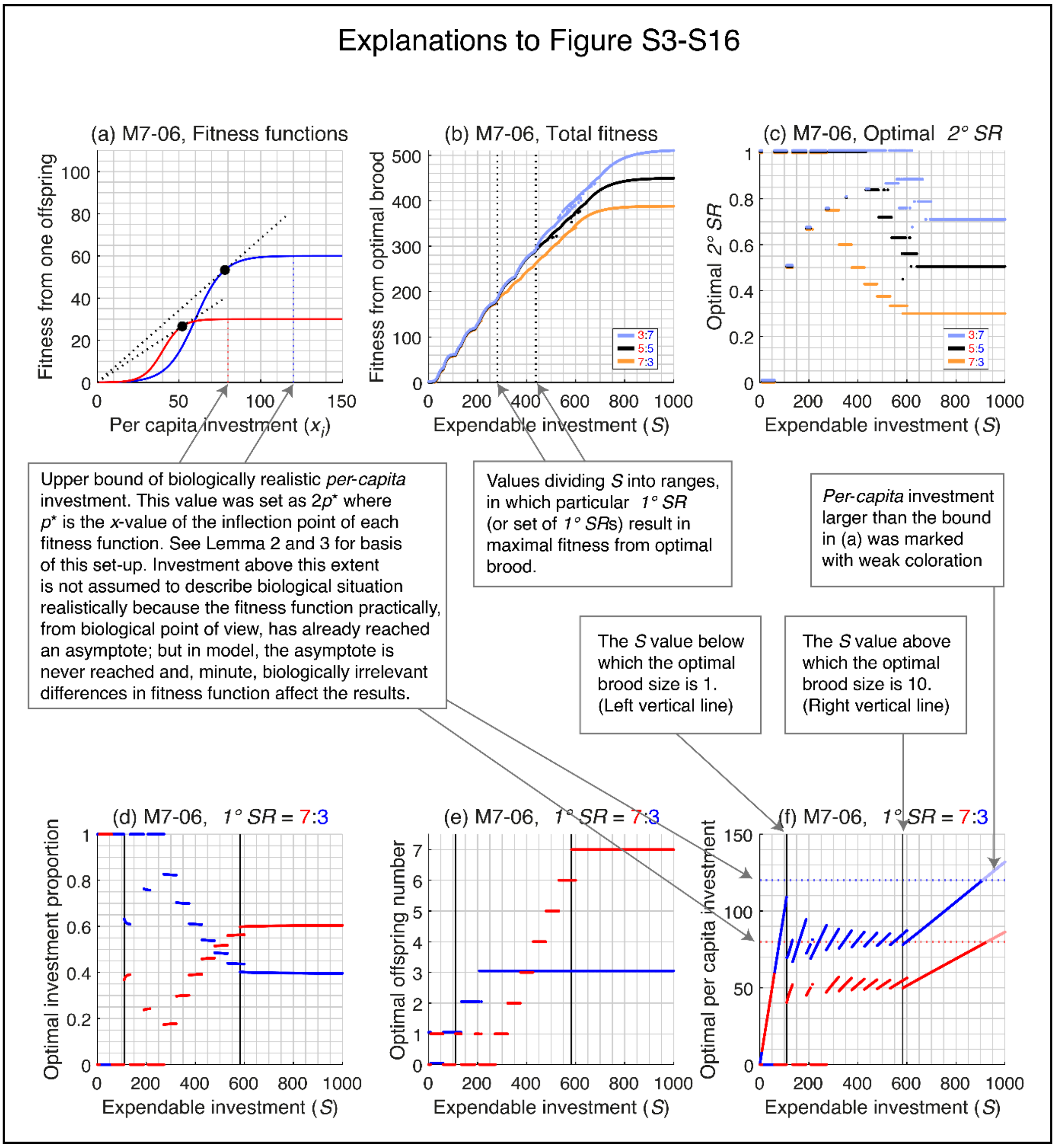

Figure S2. Graphical explanations to figures S3-S16. The schematics show how to read and interpret the panels in Figure S3-S16. Note that lines in (**b**), (**c**), (**e**), (**h**), (**k**) of Fig. S3-S16 are slightly shifted in graphs in order to avoid complete overlap of the data points.

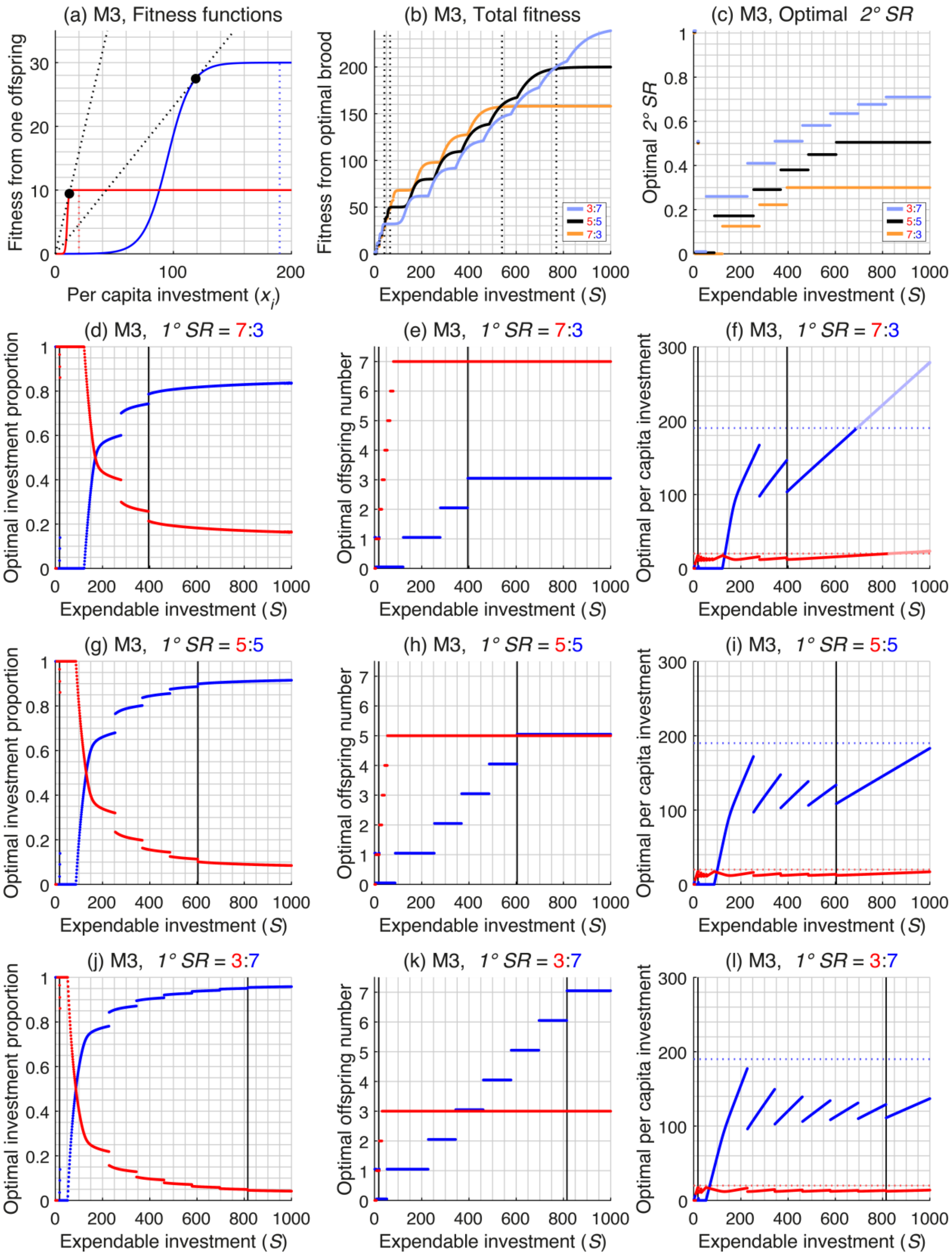

Figure S3. Summary of model *M3* (corresponding to Fig. 1e-j). **(a)** Fitness functions of *Sex 1* and *Sex 2* offspring. In this case, the slope of the tangent line is steeper for *Sex 1*. **(b)** The relationship between parental investment (S) and the fitness accrued from the full brood (sum of fitness accrued from each offspring in the brood) by optimally allocating parents of three types of broods: *Sex 1*-biased (yellow), equal (black), *Sex*-*2*-biased brood (light blue). **(c)** The relationship between S and the *2° SR* (secondary sex ratio) for optimally allocating parents in three types of broods: *Sex*-*1*-biased (yellow), equal (black), *Sex*-*2*-biased brood (light blue). **(d, g, j)** The relationships between S and the proportion of total investment given to *Sex 1* and *Sex 2* in the three types of broods: *Sex 1*-biased (d), equal (g), and *Sex*-*2*-biased (j) broods. **(e, h, k)** The relationships between S and the number of *Sex 1* and *Sex 2* offspring in a brood for the three types of broods: *Sex*-*1*-biased (e), equal (h), and *Sex*-*2*-biased (k) broods. **(f, i, l)** The relationships between S and *per capita* investment to *Sex 1* and *Sex 2* offspring in a brood for the three types of broods: *Sex*-*1*-biased (f), equal (i), and *Sex*-*2*-biased (l) broods.

J

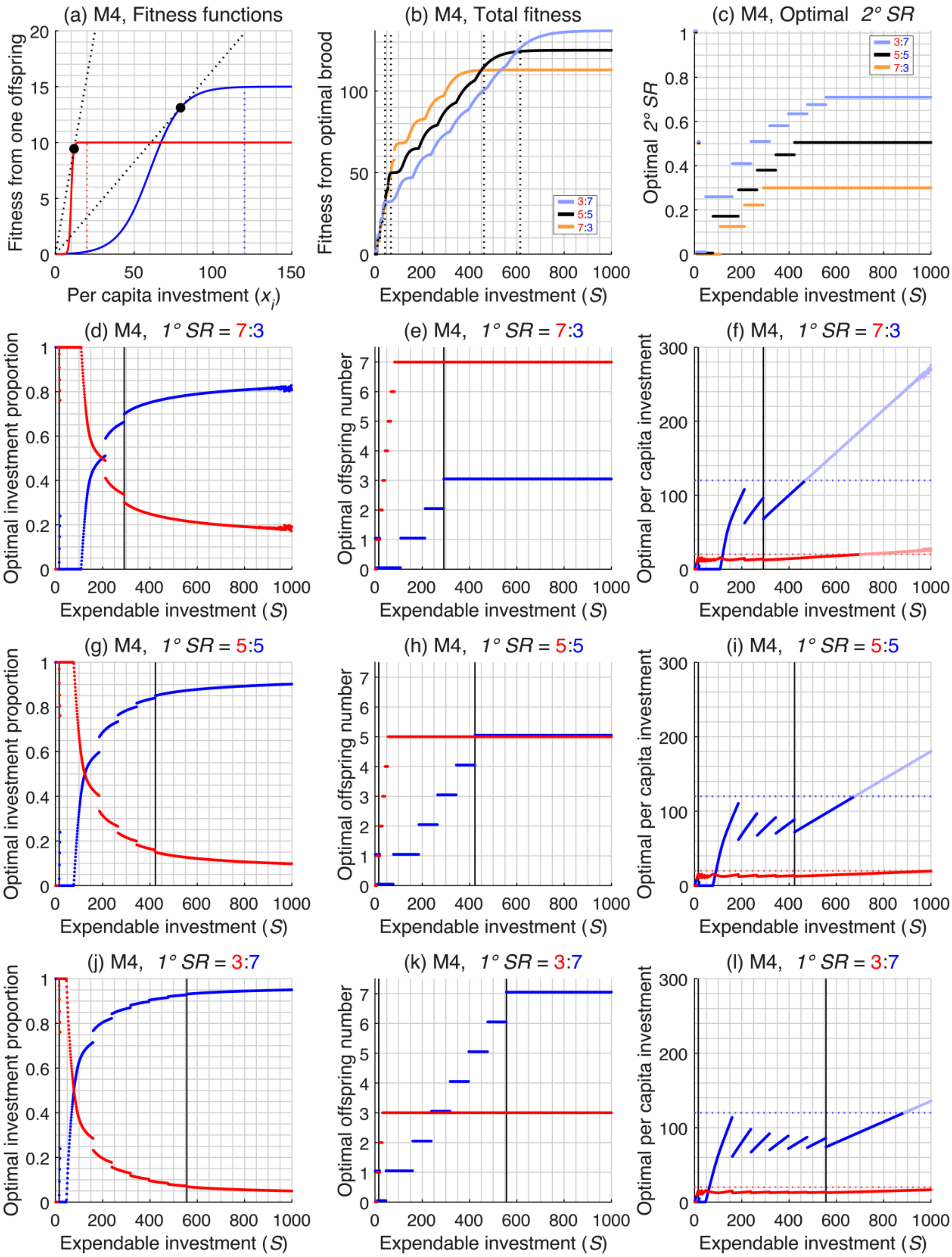

Figure S4. Summary of model *M4.* In this case, the slope of the tangent line is steeper for *Sex 1* similar to *M3* but the difference in fitness between *Sex 1* and *Sex 2* offspring is smaller than *M3*. The panels are arranged in the same manner as Fig. S3. For more explanations, see Fig. S2 and caption to Fig. S3.

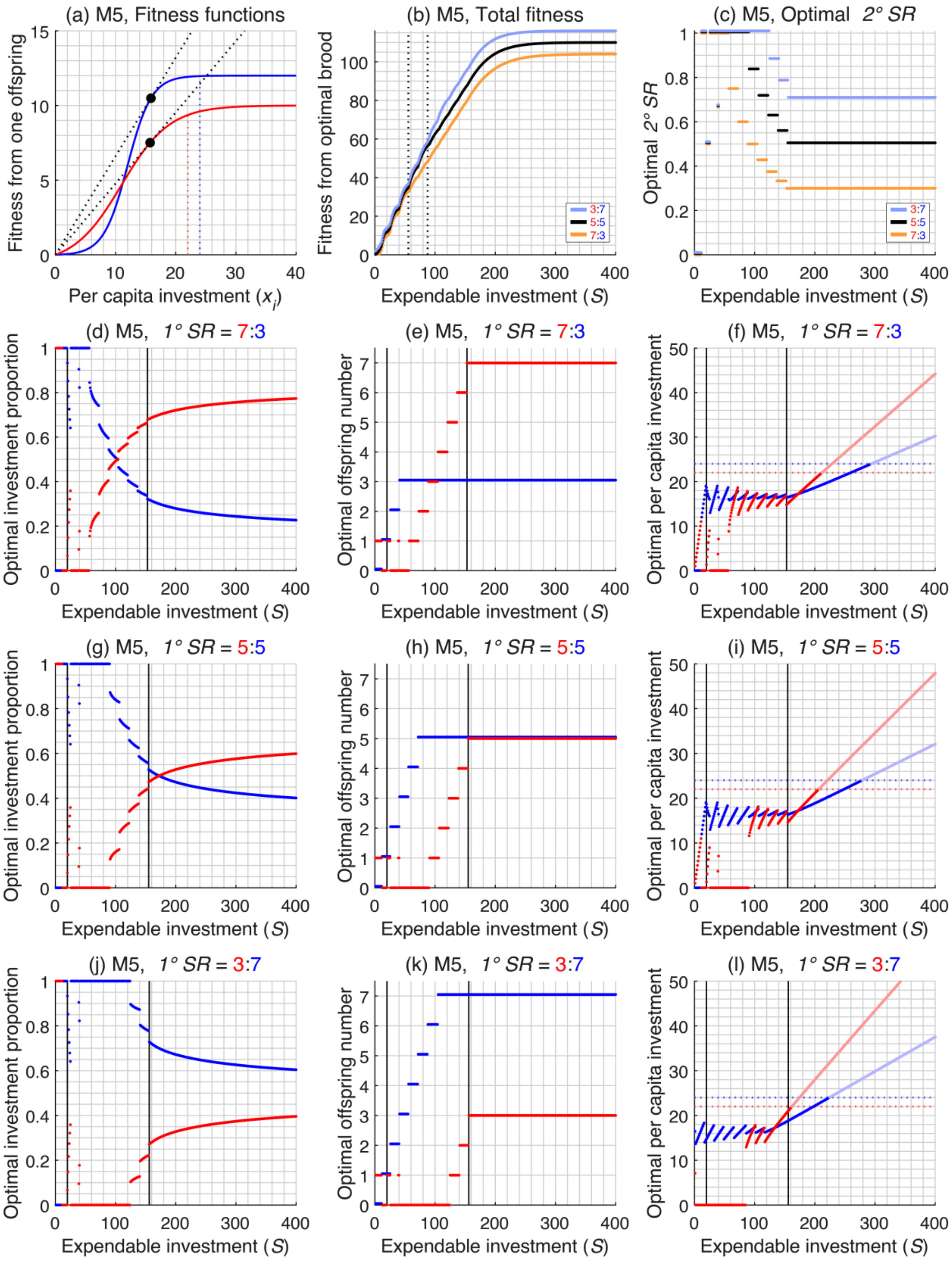

Figure S5. Summary of model *M5.* In this case, the slope of the tangent line is steeper for *Sex 2*. The panels are arranged in the same manner as Fig. S3. For more explanations, see Fig. S2 and caption to Fig. S3.

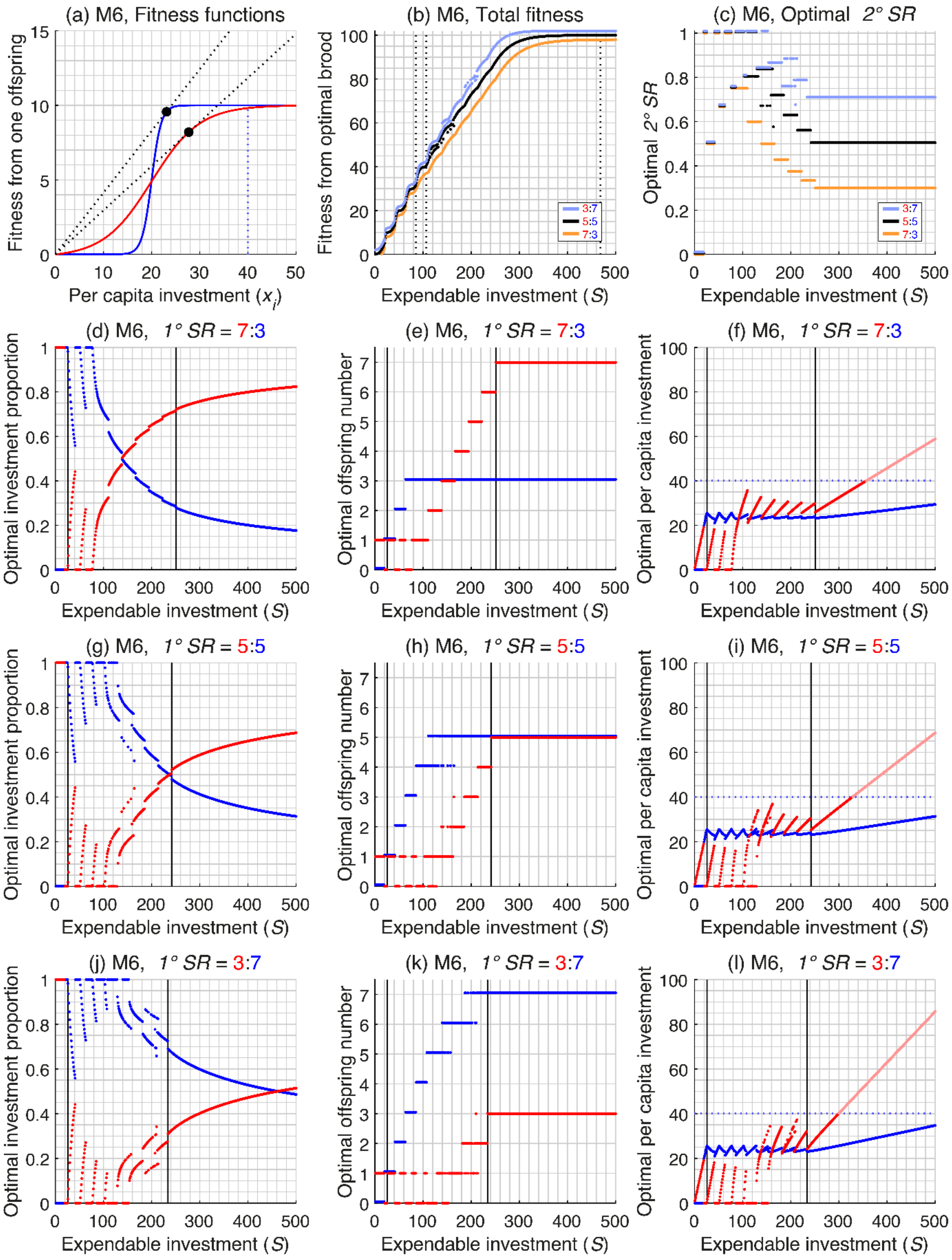

Figure S6. Summary of model *M6.* In this case, the slope of the tangent line is steeper for *Sex 2* though asymptotes are equal. The panels are arranged in the same manner as Fig. S3. For more explanations, see Fig. S2 and caption to Fig. S3.

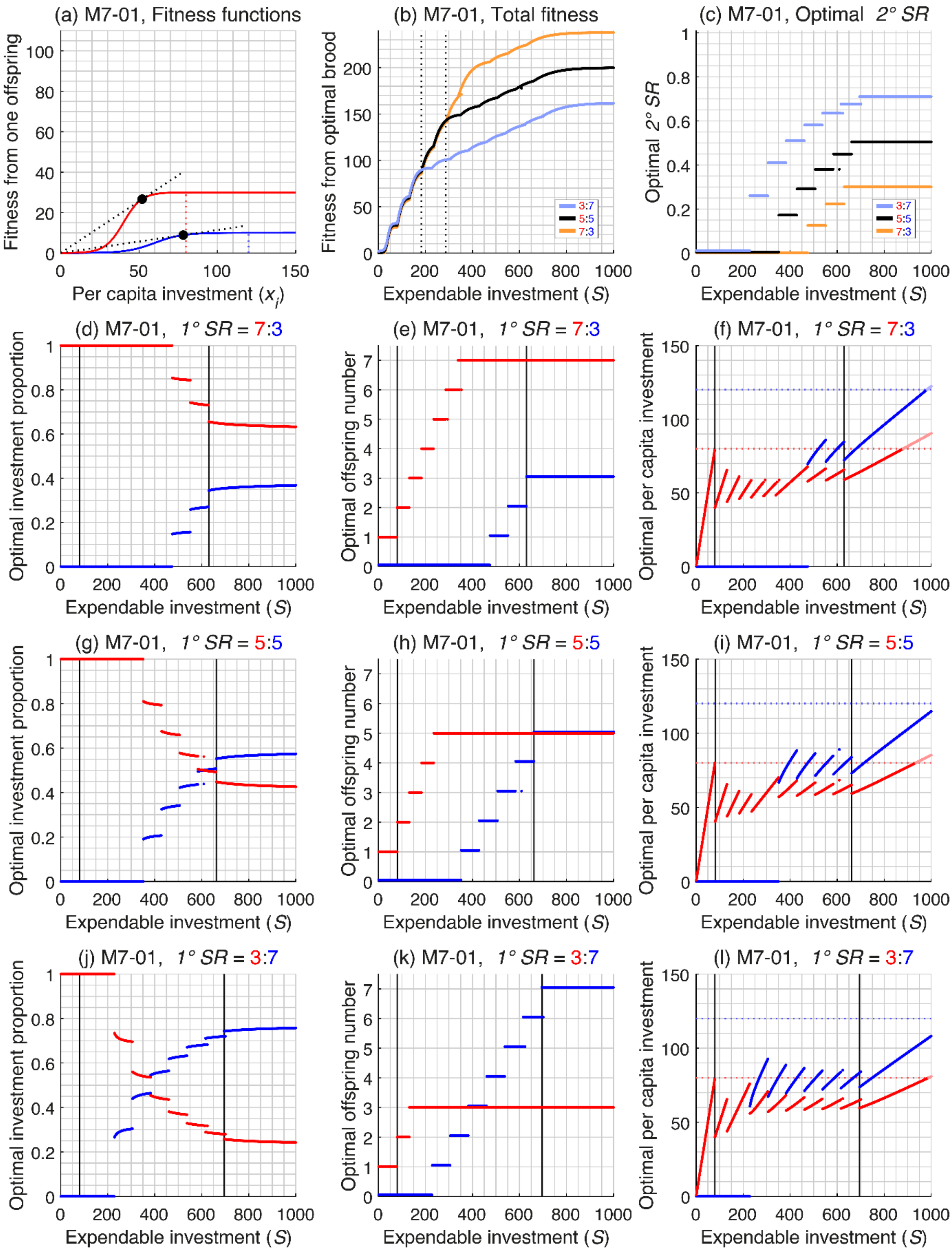

Figure S7. Summary of model *M7-1*. In this case, the slope of the tangent line is steeper for *Sex 1*. The asymptote of *Sex 1* is 30 and that of *Sex 2* is 10. The panels are arranged in the same manner as Fig. S3. For more explanations, see Fig. S2 and caption to Fig. S3.

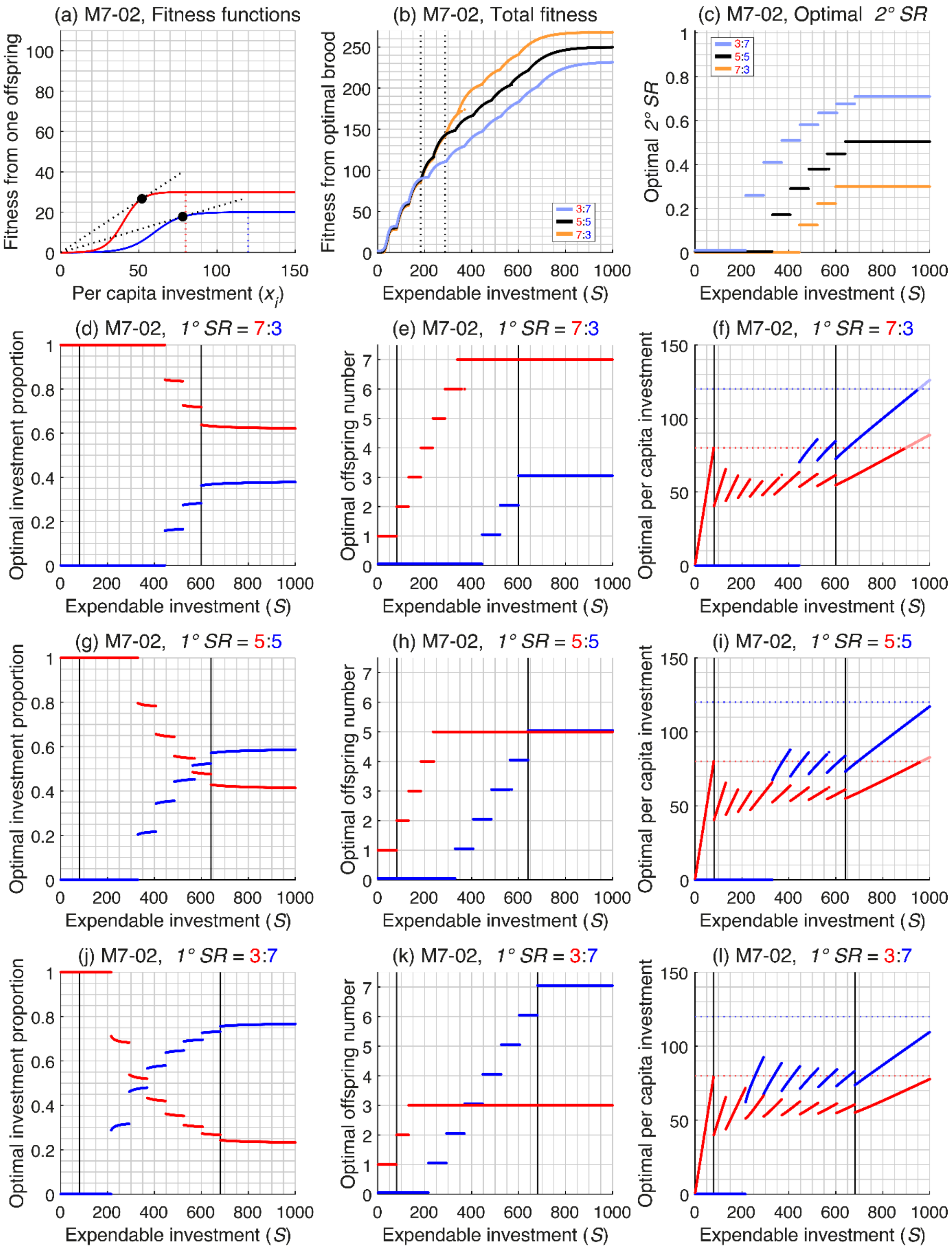

Figure S8. Summary of model *M7-2*. In this case, the slope of the tangent line is steeper for *Sex 1*. The asymptote of *Sex 1* is 30 and that of *Sex 2* is 20. The panels are arranged in the same manner as Fig. S3. For more explanations, see Fig. S2 and caption to Fig. S3.

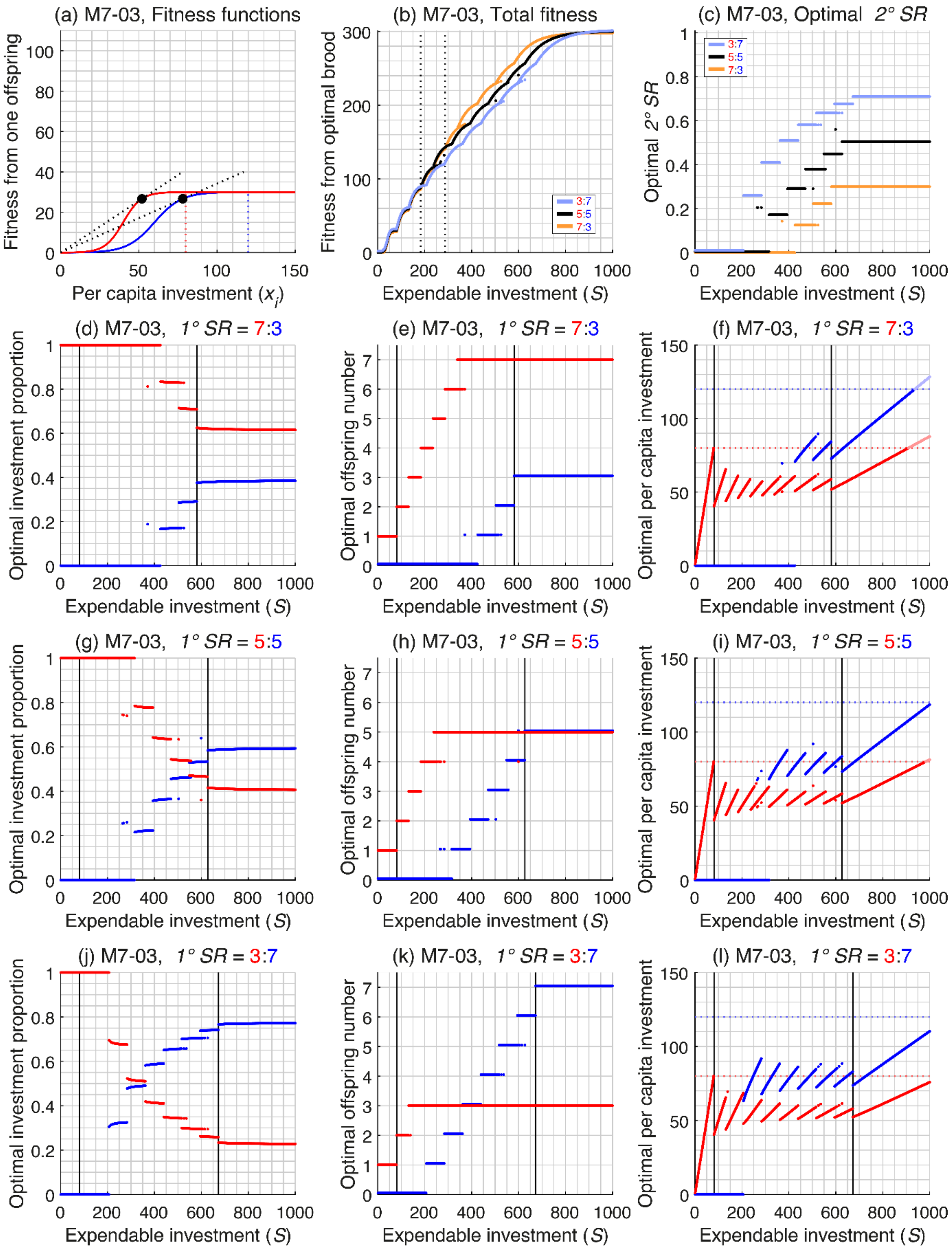

Figure S9. Summary of model *M7-3*. In this case, the slope of the tangent line is steeper for *Sex 1*. The asymptote of *Sex 1* is 30 and that of *Sex 2* is 30. The panels are arranged in the same manner as Fig. S3. For more explanations, see Fig. S2 and caption to Fig. S3.

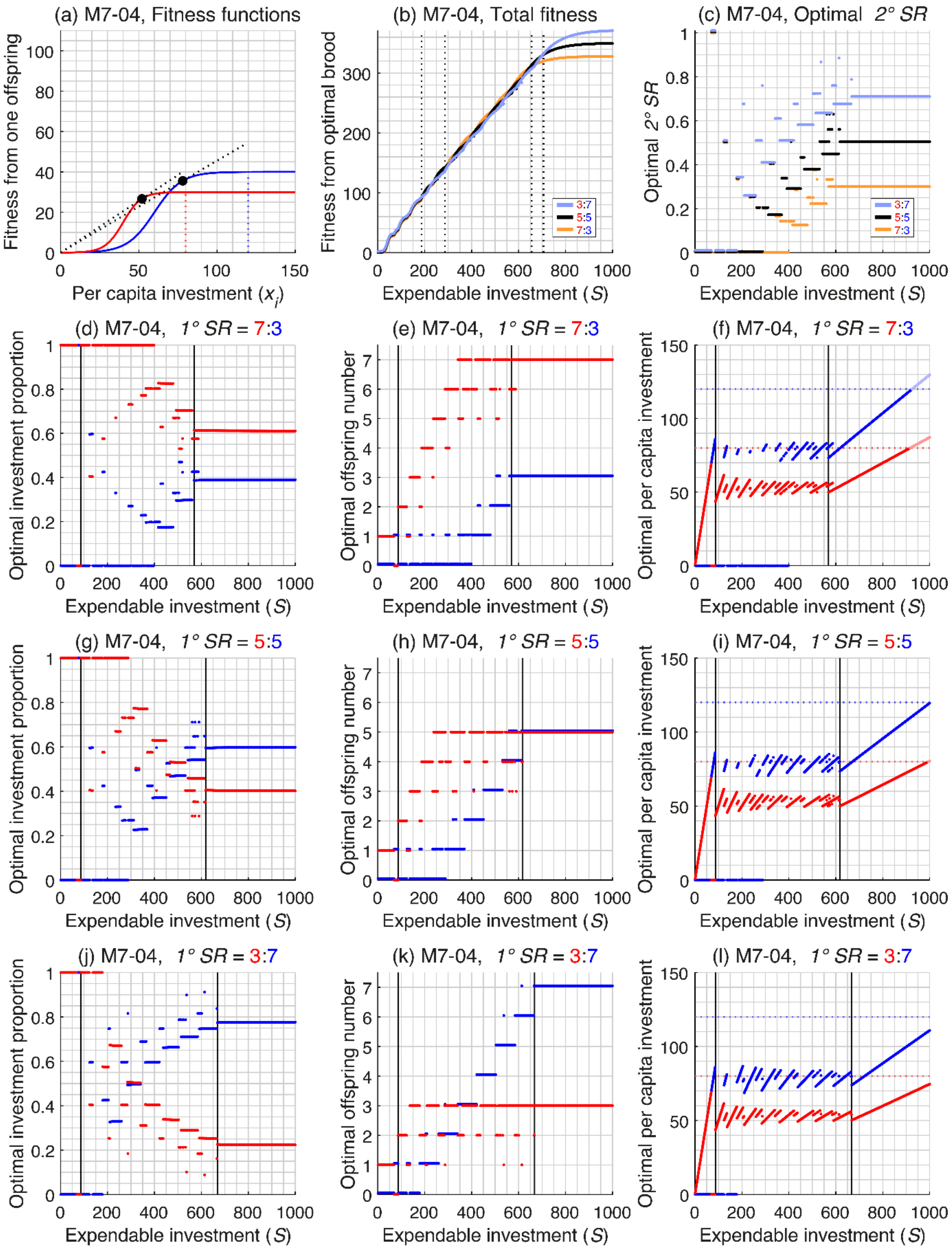

Figure S10. Summary of model *M7-4*. In this case, the slope of the tangent line is steeper for *Sex 1*. The asymptote of *Sex 1* is 30 and that of *Sex 2* is 40. The panels are arranged in the same manner as Fig. S3. For more explanations, see Fig. S2 and caption to Fig. S3.

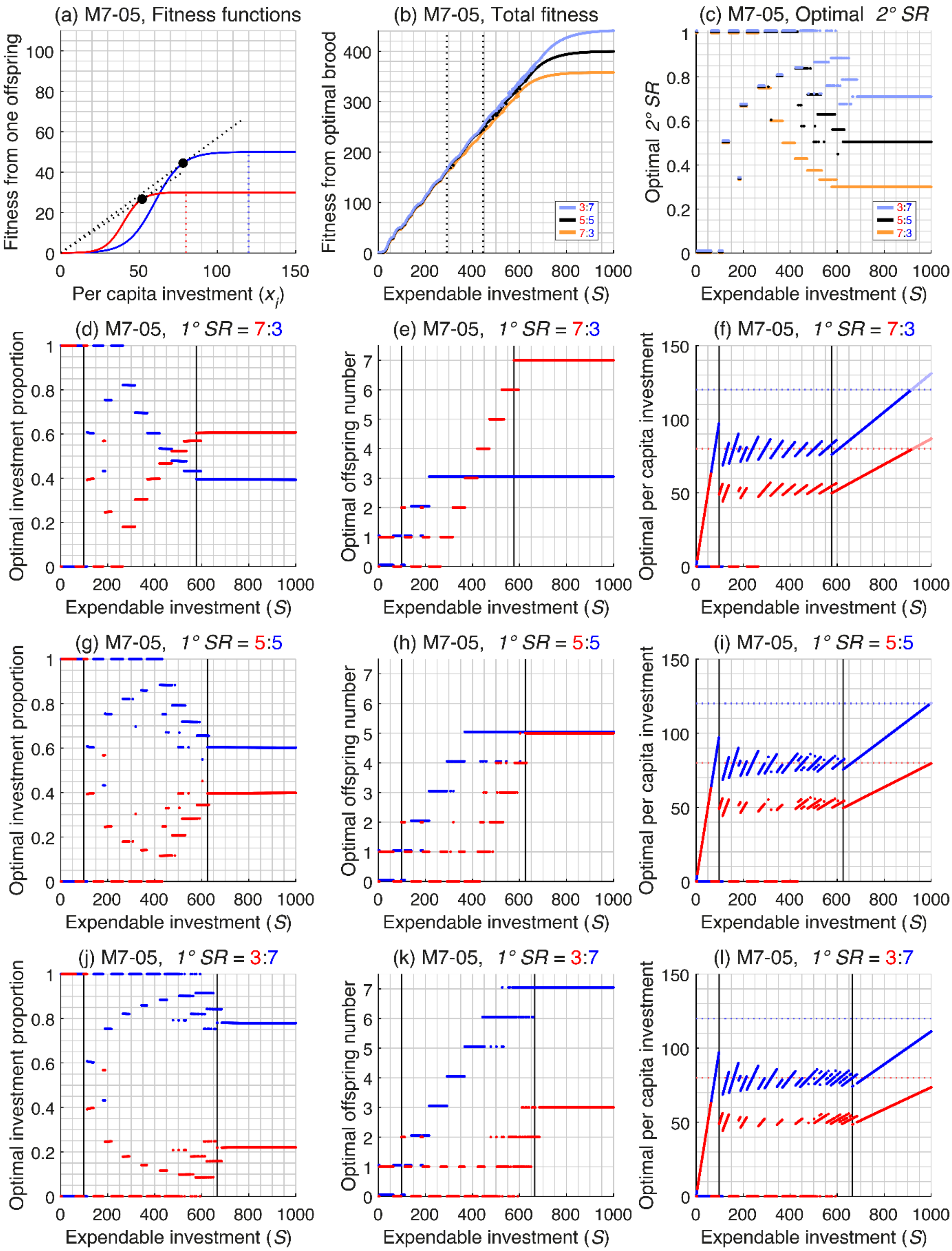

Figure S11. Summary of model *M7-5*. In this case, the slope of the tangent line is steeper for *Sex 2*. The asymptote of *Sex 1* is 30 and that of *Sex 2* is 50. The panels are arranged in the same manner as Fig. S3. For more explanations, see Fig. S2 and caption to Fig. S3.

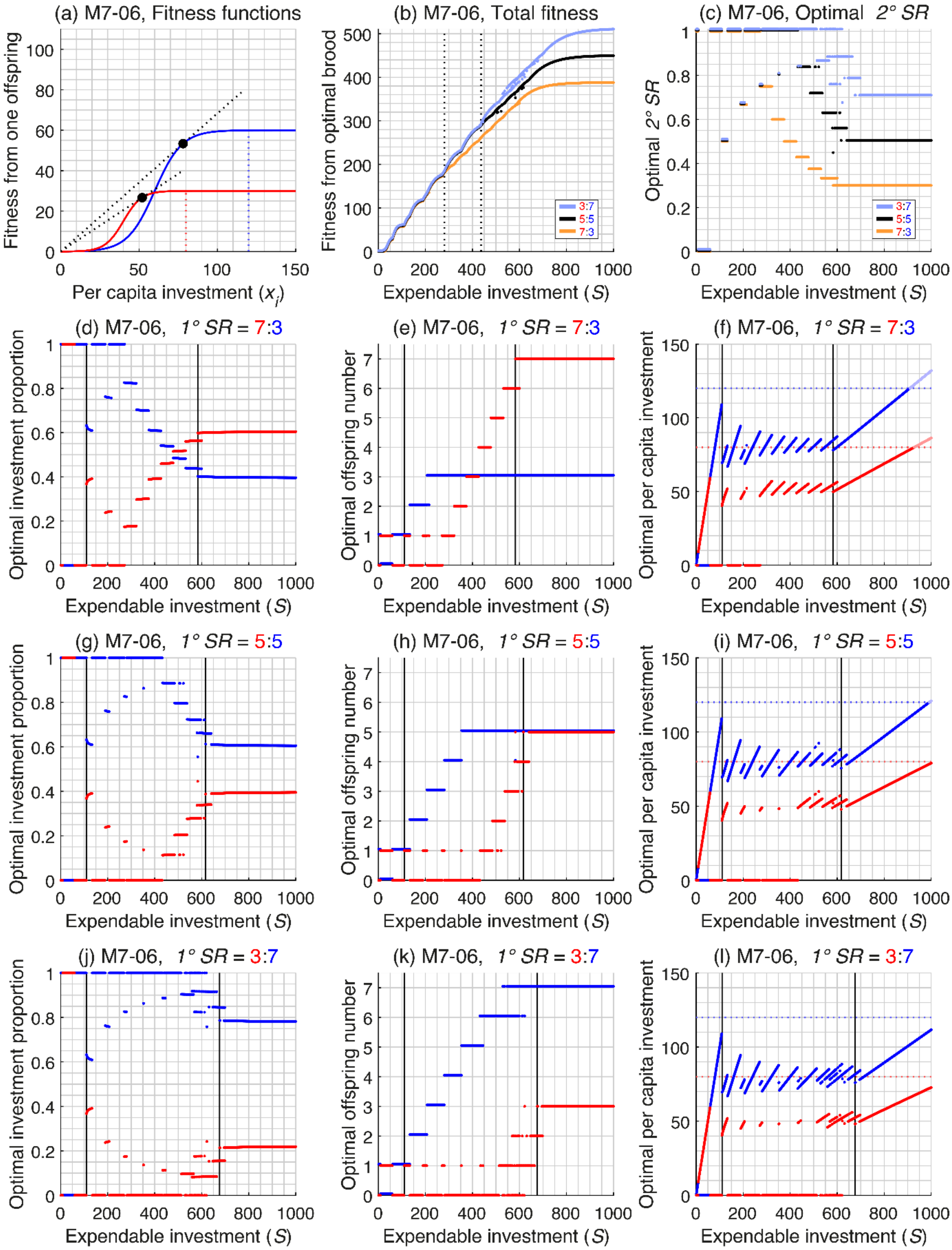

Figure S12. Summary of model *M7-6*. In this case, the slope of the tangent line is steeper for *Sex 2*. The asymptote of *Sex 1* is 30 and that of *Sex 2* is 60. The panels are arranged in the same manner as Fig. S3. For more explanations, see Fig. S2 and caption to Fig. S3.

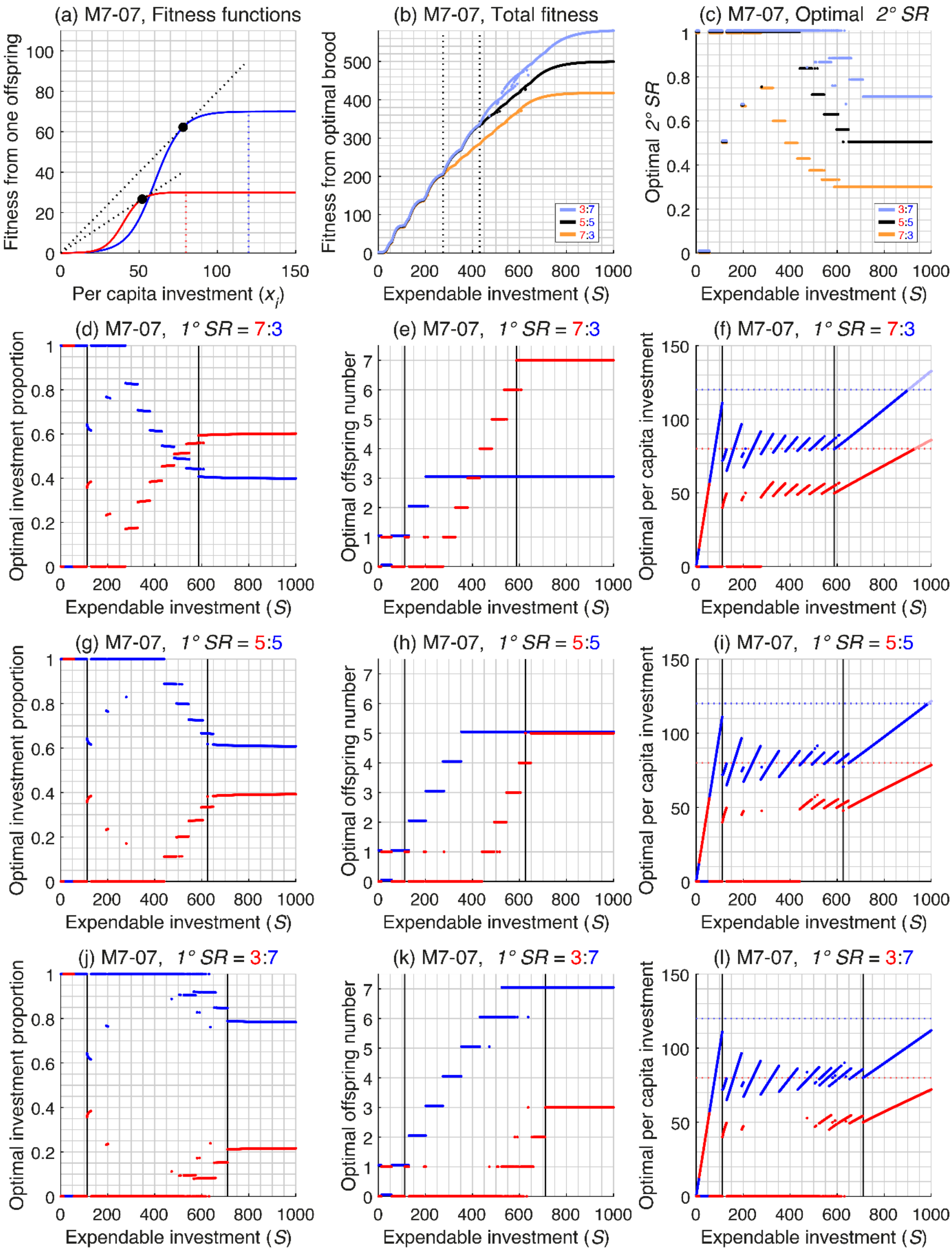

Figure S13. Summary of model *M7-7*. In this case, the slope of the tangent line is steeper for *Sex 2*. The asymptote of *Sex 1* is 30 and that of *Sex 2* is 70. The panels are arranged in the same manner as Fig. S3. For more explanations, see Fig. S2 and caption to Fig. S3.

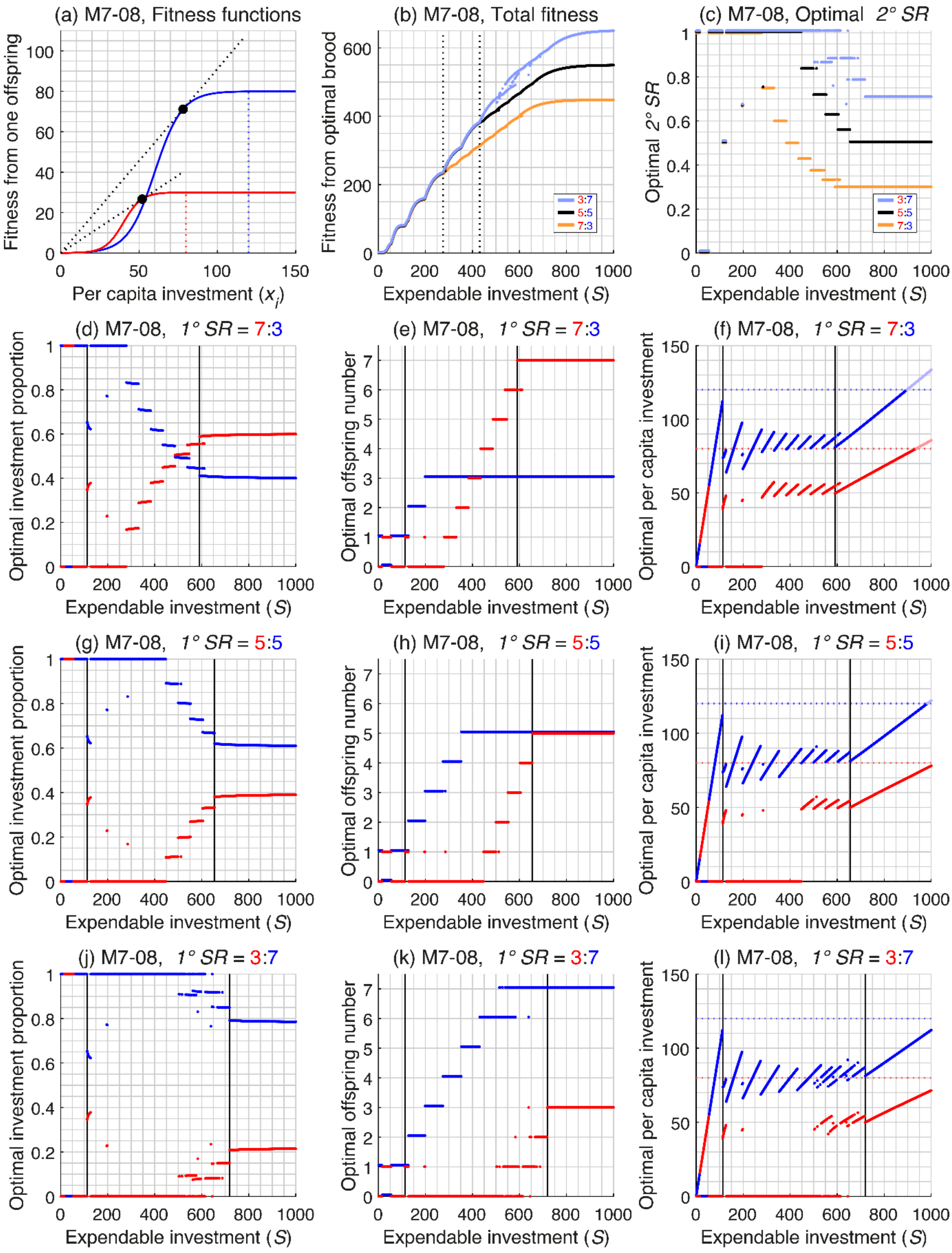

Figure S14. Summary of model *M7-8*. In this case, the slope of the tangent line is steeper for *Sex 2*. The asymptote of *Sex 1* is 30 and that of *Sex 2* is 80. The panels are arranged in the same manner as Fig. S3. For more explanations, see Fig. S2 and caption to Fig. S3.

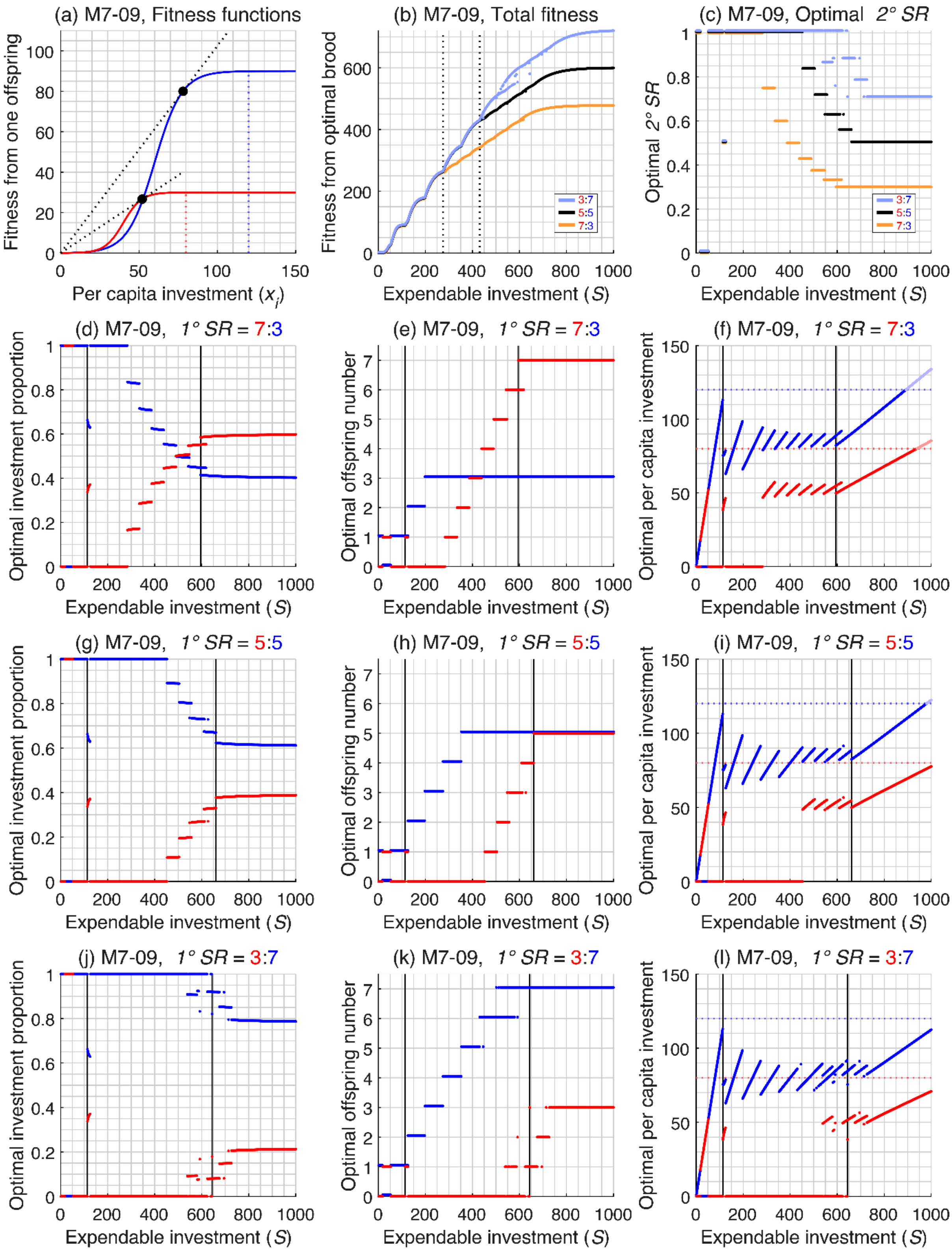

Figure S15. Summary of model *M7-9*. In this case, the slope of the tangent line is steeper for *Sex 2*. The asymptote of *Sex 1* is 30 and that of *Sex 2* is 90. The panels are arranged in the same manner as Fig. S3. For more explanations, see Fig. S2 and caption to Fig. S3.

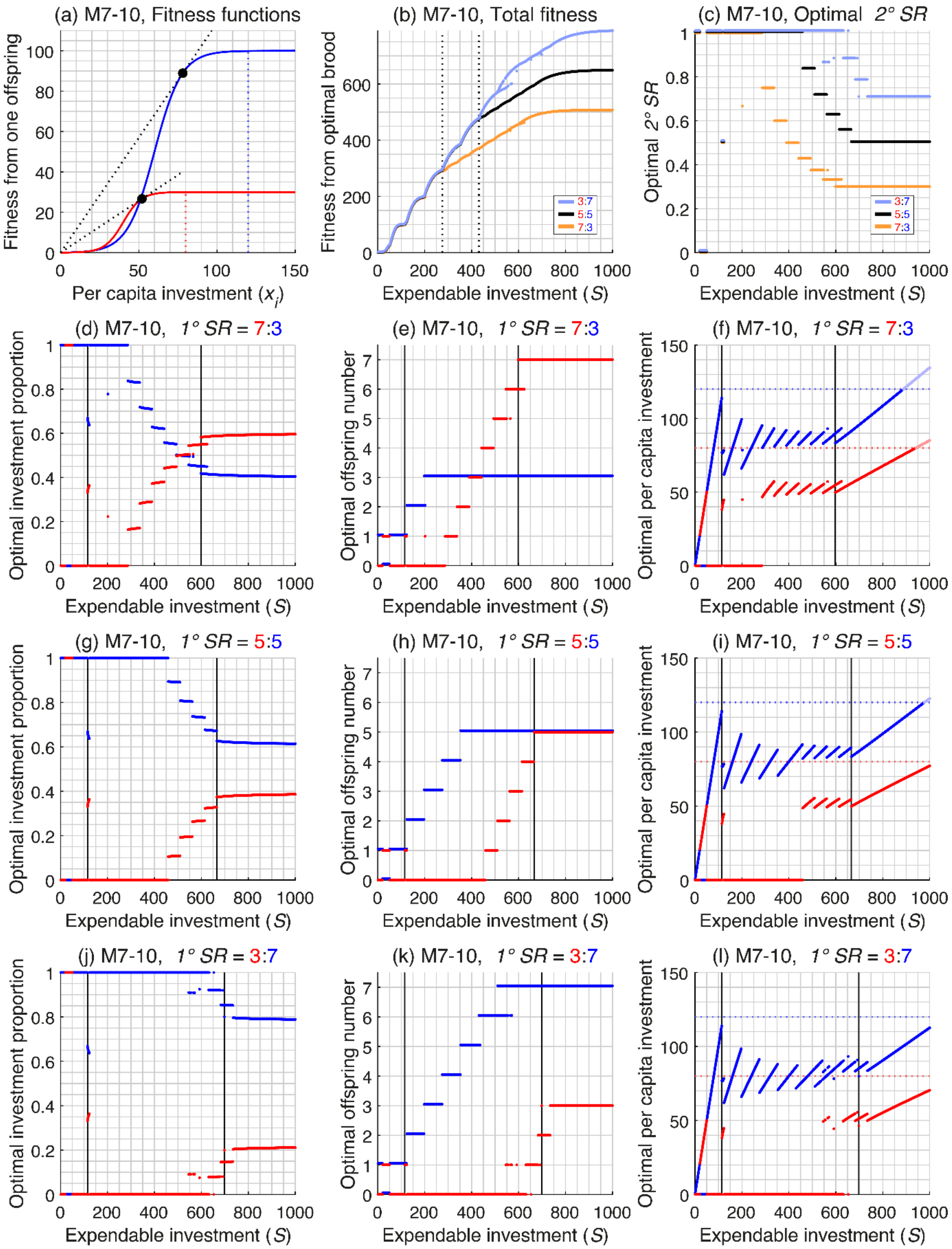

Figure S16. Summary of model *M7-10*. In this case, the slope of the tangent line is steeper for *Sex 2*. The asymptote of *Sex 1* is 30 and that of *Sex 2* is 100. The panels are arranged in the same manner as Fig. S3. For more explanations, see Fig. S2 and caption to Fig. S3.

Summary of locally optimal strategies

Table S2. Comparison of locally optimal strategies with the predictions from the classical TWH. Predictions from the classical TWH are solely dependent on the variance (range) of each fitness function. The table concerns Fig. S3-S16. It was defined that one sex is 'favored' over another if the proportion of *S* invested in the former sex is larger than 50%. The pattern of the proportion of *S* (panels (d), (g), (j) in Fig. S3-S16) is regarded to be fully consistent with the classical TWH if a change of proportion of *S* from <50% to >50% occurs for the sex with larger variation (range) in the fitness function. The pattern of *per capita* investment (panels (f), (i), (l) in Fig. S3-S16) is regarded to be consistent with the classical TWH if, for larger *S*, the *per capita* investment (x_i) of the sex with the larger range in fitness function is higher than that of another sex. All other situations are classified to be inconsistent with the classical TWH. These include the reversed TWH, defined as a change of proportion of *S* from <50% to >50% for the sex with smaller variation (range) in the fitness function.

	**The general pattern of locally optimal strategy**		**Prediction from classical TWH**	**Conformity between TWH predictions and model results**	
Model name/ figure number	***Per capita* investment**: Sex with the greater *per capita* investment for a range of high *S* (n*=10, but *S*≤1000, based on panels (f), (i), (l))	**Proportion of total Investment**: Sex with the increasing proportion of *S* as *S* increases and possibly leading to the proportion of *S* >50% for high *S* (n*=10, but *S*≤1000, based on panels (d), (g), (j))	Sex that should become 'favored' under TWH when *S* increases	*Per capita* investment	The proportion of total investment
M3/ S3	*Sex 2*	*Sex 2*	*Sex 2*	Consistent	Consistent
M4/ S4	*Sex 2*	*Sex 2*	*Sex 2*	Consistent	Consistent
M5/ S5	*Sex 1*	Investment in *Sex 1* increases with increasing *S*, but only in 7:3 and 5:5; broods become >50% of *S*. Investment in *Sex 2* always >50% of *S* in 3:7 broods	*Sex 2*	Inconsistent	Inconsistent in all broods; reversed TWH in broods 7:3 and 5:5
M6/ S6	*Sex 1*	*Sex 1*	No favored sex	Inconsistent	Inconsistent
M7-1/ S7	*Sex 2*	*Sex 1* in 7:3; but *Sex 2* in 3:7 and 5:5 broods	*Sex 1*	Inconsistent	Inconsistent for 7:3 and 5:5 broods
M7-2/ S8	*Sex 2*	*Sex 1* in 7:3; but *Sex 2* in 3:7 and 5:5 broods	*Sex 1*	Inconsistent	Inconsistent for 7:3 and 5:5 broods
M7-3/ S9	*Sex 2*	*Sex 1* in 7:3; but *Sex 2* in 3:7 and 5:5 broods	No favored sex	Inconsistent	Inconsistent
M7-4/ S10	*Sex 2*	*Sex 1* in 7:3; but *Sex 2* in 3:7 and 5:5 broods	*Sex 2*	Consistent	Inconsistent for 3:7 broods
M7-5/ S11	*Sex 2*	*Sex 1* in 7:3; but *Sex 2* in 3:7 and 5:5 broods	*Sex 2*	Consistent	Inconsistent for 3:7 broods
M7-6/ S12	*Sex 2*	*Sex 1* in 7:3; but *Sex 2* in 3:7 and 5:5 broods	*Sex 2*	Consistent	Inconsistent for 3:7 broods
M7-7/ S13	*Sex 2*	*Sex 1* in 7:3; but *Sex 2* in 3:7 and 5:5 broods	*Sex 2*	Consistent	Inconsistent for 3:7 broods
M7-8/ S14	*Sex 2*	*Sex 1* in 7:3; but *Sex 2* in 3:7 and 5:5 broods	*Sex 2*	Consistent	Inconsistent for 3:7 broods
M7-9/ S15	*Sex 2*	*Sex 1* in 7:3; but *Sex 2* in 3:7 and 5:5 broods	*Sex 2*	Consistent	Inconsistent for 3:7 broods
M7-10/ S16	*Sex 2*	*Sex 1* in 7:3; but *Sex 2* in 3:7 and 5:5 broods	*Sex 2*	Consistent	Inconsistent for 3:7 broods

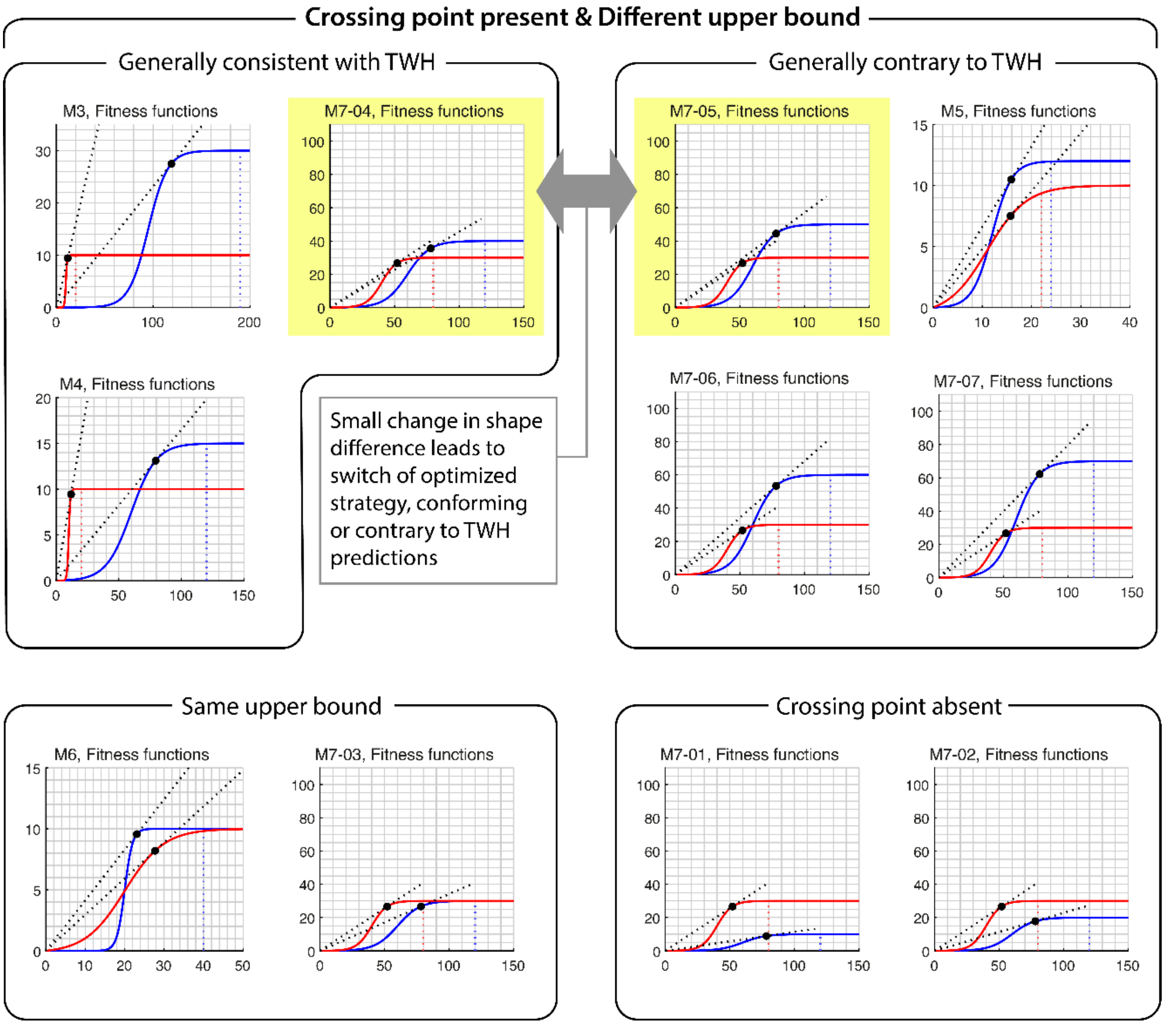

Figure S17. Classification of pairs of fitness functions with regard to the shape differences between the fitness functions ('crossing point present & different upper bound,' 'same upper bound,' 'crossing point absent'). Locally optimal investment strategy patterns into those generally consistent with the classical TWH (left side) and those generally contrary to TWH (right side) are the two subcategories of 'crossing point present & different upper bound.' We believe that in the real world, the two sexes will often differ in the fitness function shape and that the difference involves a crossing point between the two functions. This condition indicates that one sex gains fitness faster but reaches a lower asymptote as parental investment increases, and the other sex gains fitness slower but reaches higher maximal fitness as parental investment increases. Models *M7-8*, *M7-9*, *M7-10* are not shown here due to the space constraints and they belong to the 'generally contrary to TWH' category. Yellow shading and arrows indicate two models *(M7-04* and *M7-05*) that differ slightly in the fitness function shape. This difference is sufficient to create a change of the sex with the steeper slope of the tangent line. Hence, these two models illustrate the reversal from the classical TWH to the reversed TWH predictions, which is also illustrated in the Supplementary Animation S1 and S2.

The patterns observed in panels (e), (h), (k) of Fig. S3-S16 (also Fig 1i, o) may suggest that the tangent-line rule of thumb is also applicable to the general pattern of the number of offspring for each sex and to the resultant locally (panels (c) in Fig. S3-S16) and globally optimal (panels (c) in Fig. S19-S32) pattern of secondary sex ratio, but there is an evident counterexample (Fig. S34 and Remark 3 in Part 4). In this counterexample where the fitness functions extremely differ, the tangent rule does not predict the pattern of the offspring number of each sex, while still correctly predicts the general trends in the proportion of *S* invested in each sex.

Globally optimal strategies

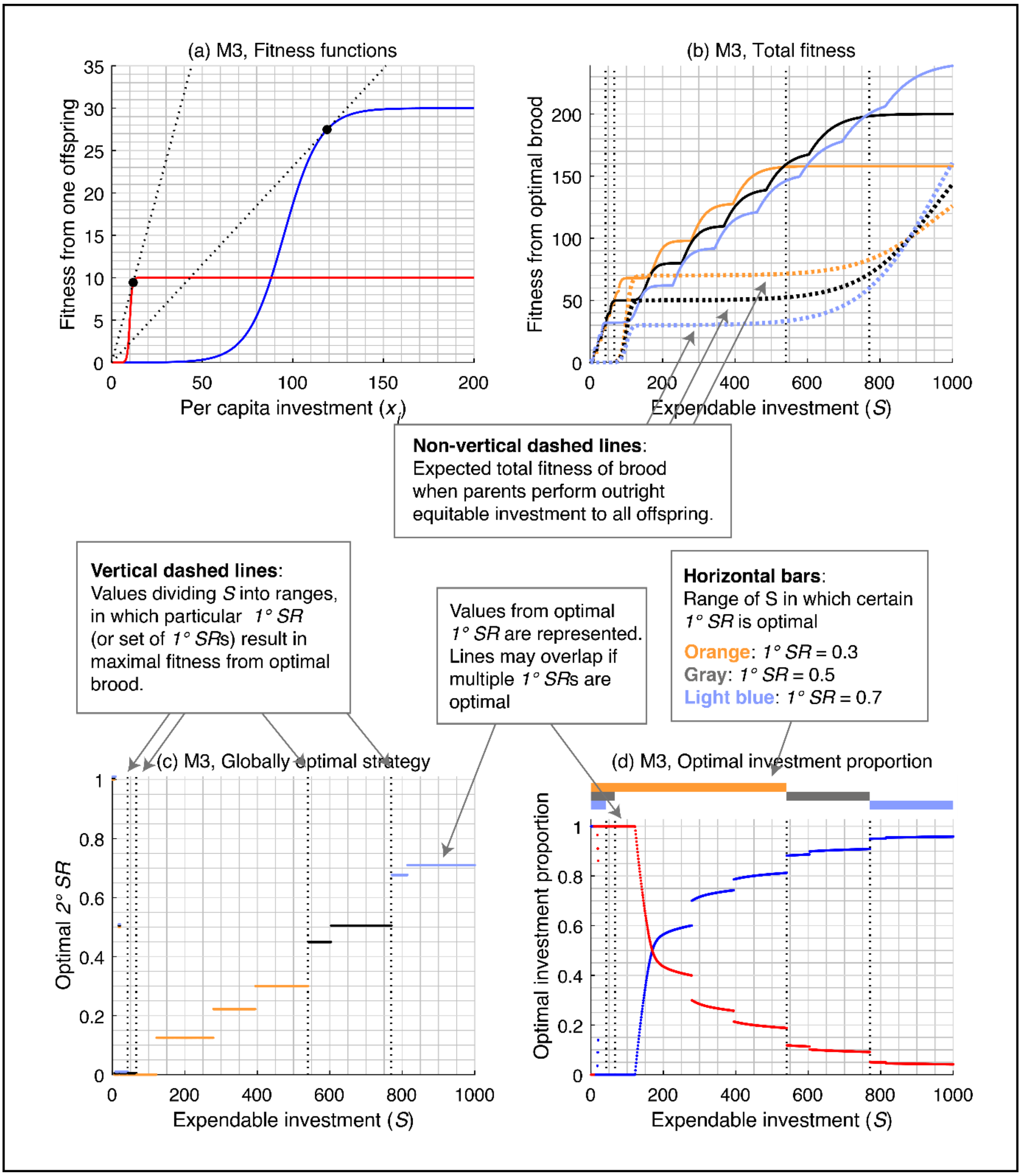

Figure S18. Graphical explanations to figures S19-S32 representing globally optimal strategies. The schematics show how to read and interpret the panels. Note that lines in (b), (c) of Fig. S19-S32 are slightly shifted in graphs in order to avoid complete overlap of the points.

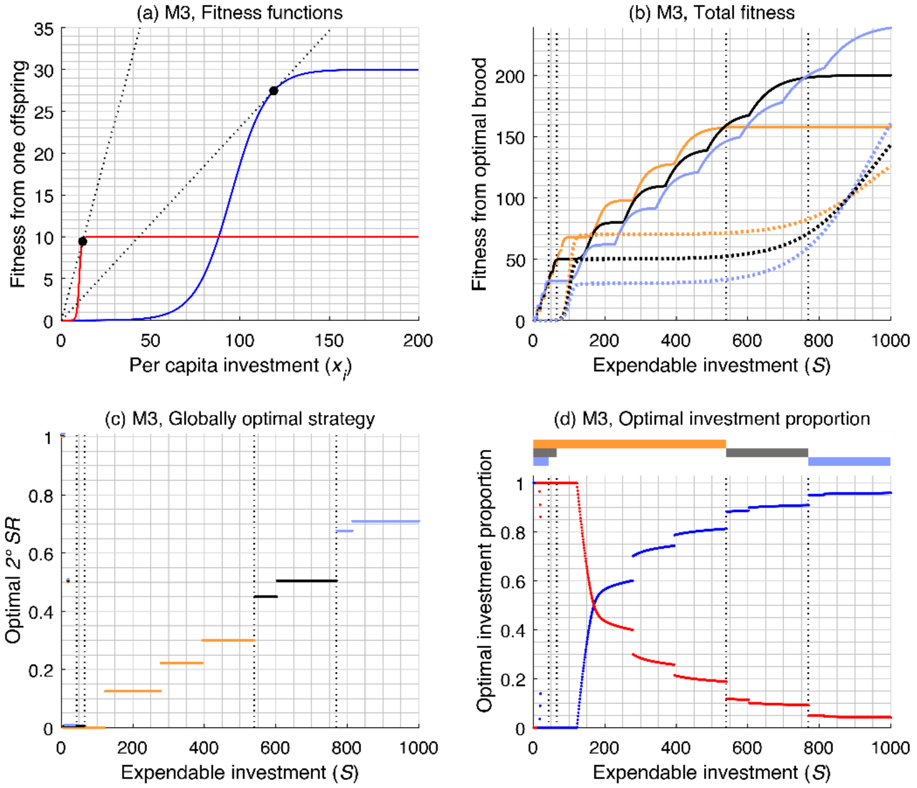

Figure S19. Fitness functions and the corresponding globally optimal strategies of model *M3*. **(a)** Fitness functions of *Sex 1* and *Sex 2* offspring. **(b)** Comparison of parental fitness for three *1° SR*s (7:3, 5:5, 3:7) between parents that optimally allocate investment (solid lines) with parents that equitably distribute the resources (dotted lines). Vertical dotted lines represent *S* where the optimal *1° SR* changes for optimally investing parents. **(c)** Schematic representations of the globally optimal *2° SR*s (represented on the *y*-axis as the proportion of *Sex 2* in the brood). These schematics are obtained by extracting the optimal *2° SR*s against *S* for globally optimal *1° SR*s from panel (c) in Fig. S3-S16. Globally optimal *1° SR* is equivalent to the fitness-maximizing *1° SR* in panel (b) of Fig. S3-S16. For example, within the interval of 66<*S*<540, *Sex*-*1*-biased brood is solely optimal as shown in (b), so that optimal *2° SR*s of the *Sex*-*1*-biased brood is shown as orange lines in panel (c). Full data is shown in (c) of Fig. S3-S16. **(d)** Schematic representations of the globally optimal investment proportion. These schematics are obtained by extracting optimal proportion against *S* for optimal *1° SR*s from panels (d), (g), (j) in Fig. S3-S16. Color-coded horizontal bars above the panel indicate the *1° SR*s included in globally optimal strategy: *Sex*-*1*-biased (yellow), equal (black), *Sex*-*2*-biased brood (light blue). For more explanations, see Fig. S18.

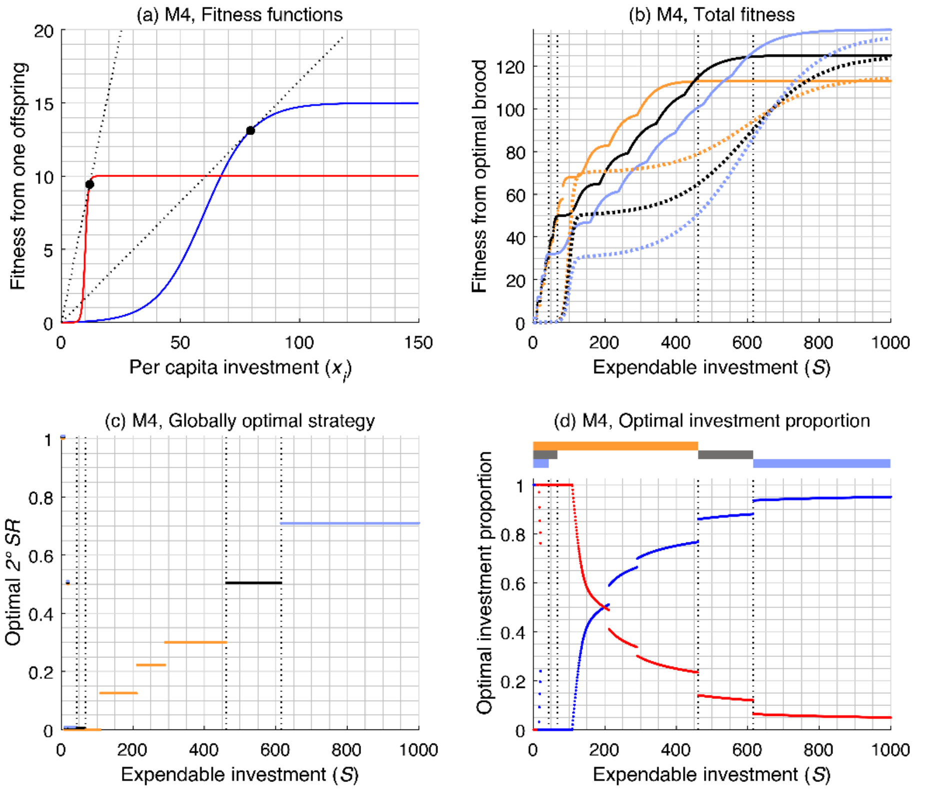

Figure S20. Fitness functions and corresponding globally optimal strategies of model *M4*. Panels are arranged as in Fig. S19.

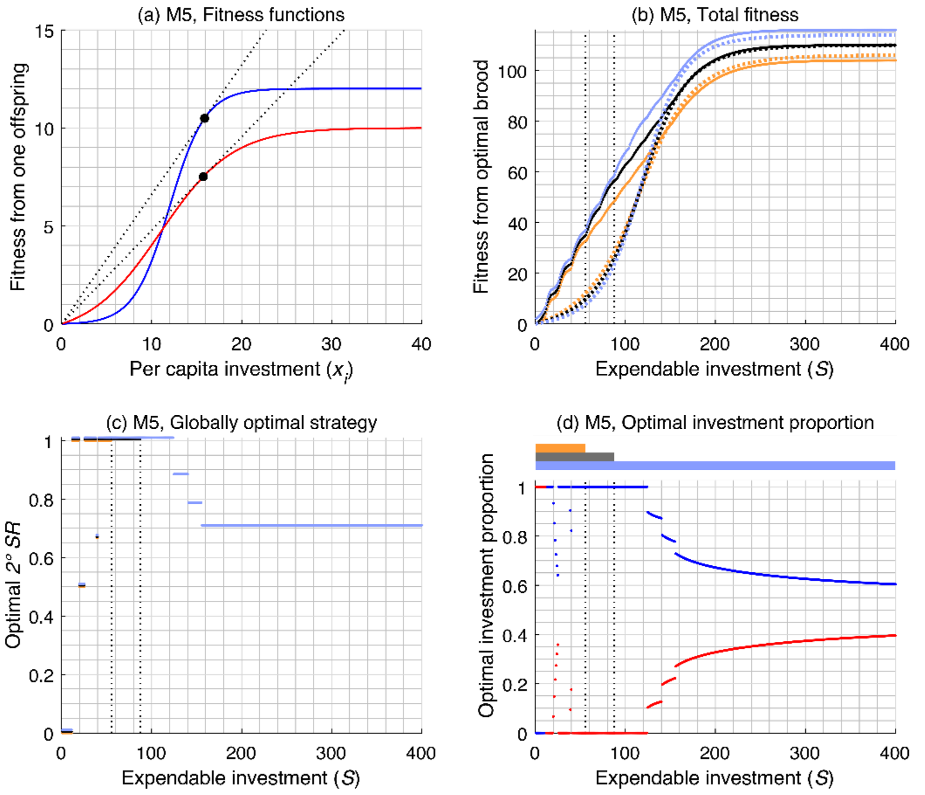

Figure S21. Fitness functions and corresponding globally optimal strategies of model *M5*. Panels are arranged as in Fig. S19. For more explanations see Fig. S18 and caption to Fig. S19.

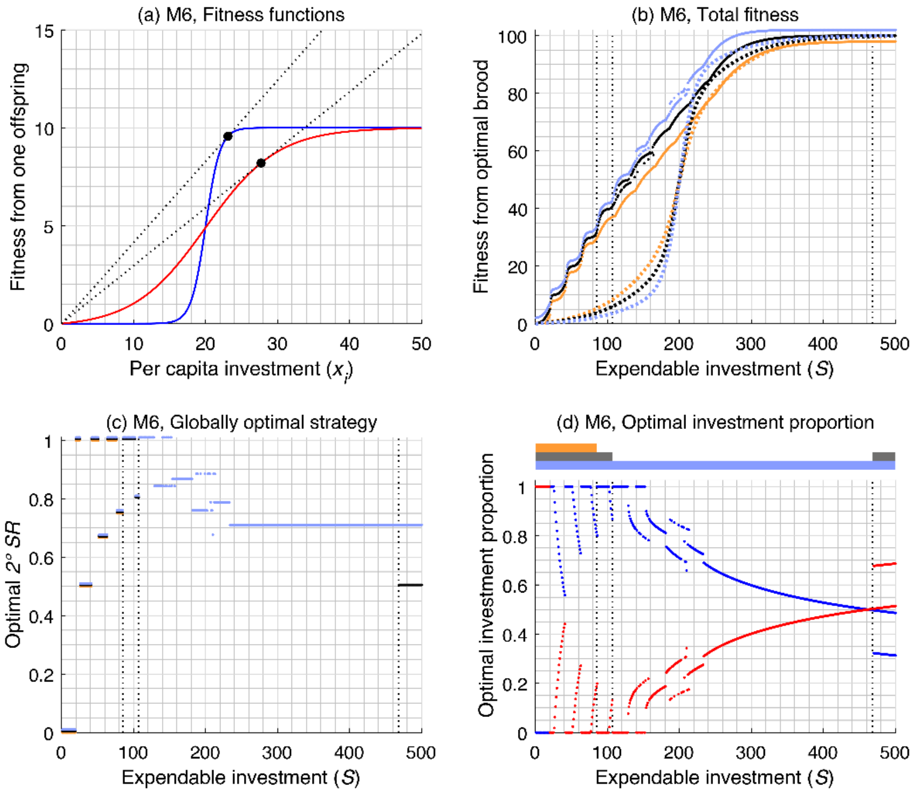

Figure S22. Fitness functions and corresponding globally optimal strategies of model *M6*. Panels are arranged as in Fig. S19. For more explanations, see Fig. S18 and caption to Fig. S19.

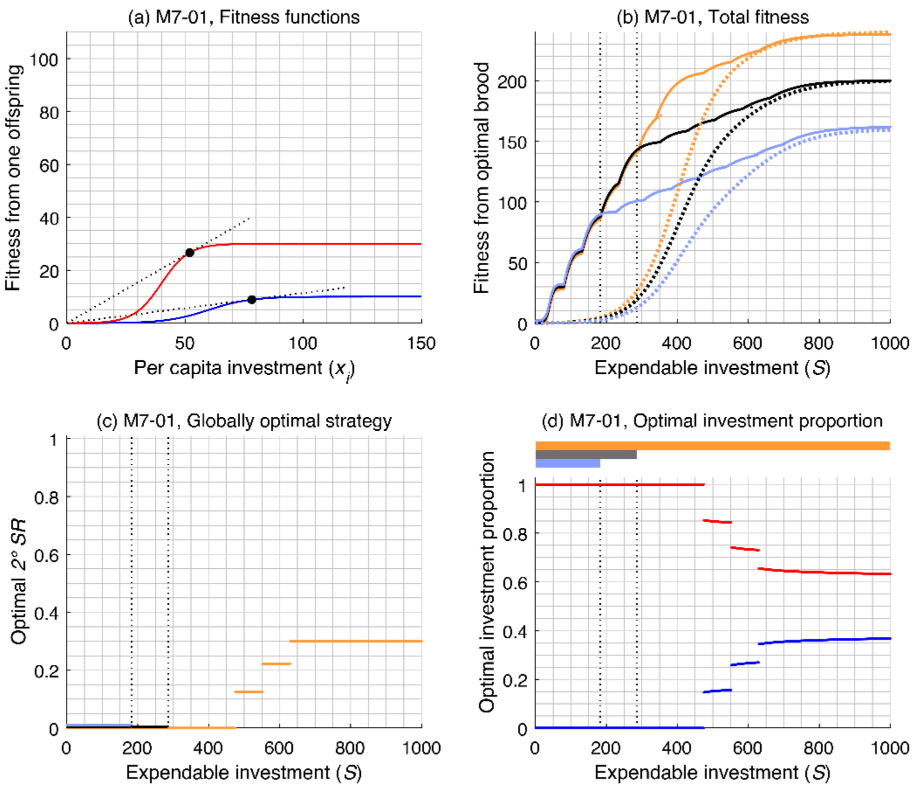

Figure S23. Fitness functions and corresponding globally optimal strategies of model *M7-1*. Panels are arranged as in Fig. S19. For more explanations, see Fig. S18 and caption to Fig. S19.

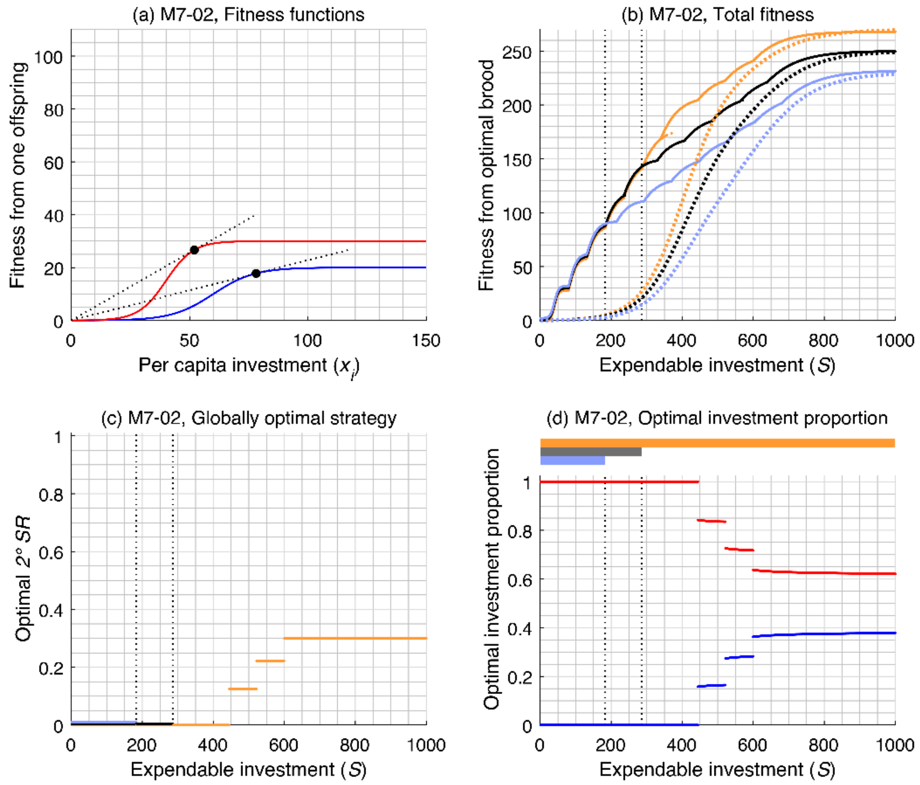

Figure S24. Fitness functions and corresponding globally optimal strategies of model *M7-2*. Panels are arranged as in Fig. S19. For more explanations, see Fig. S18 and caption to Fig. S19.

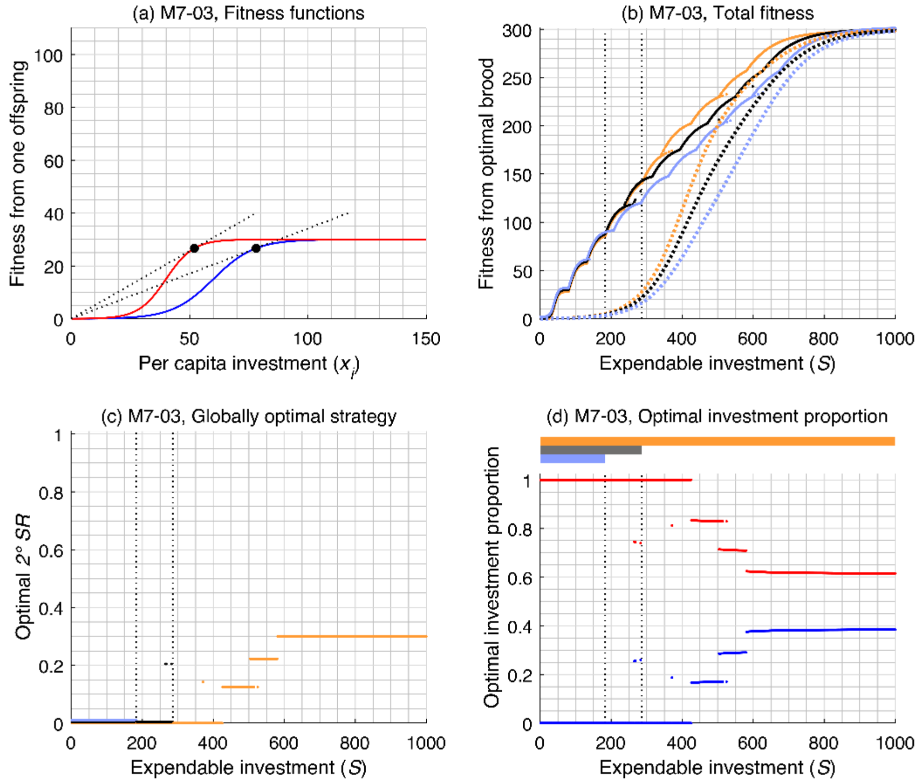

Figure S25. Fitness functions and corresponding globally optimal strategies of model *M7-3*. Panels are arranged as in Fig. S19. For more explanations, see Fig. S18 and caption to Fig. S19.

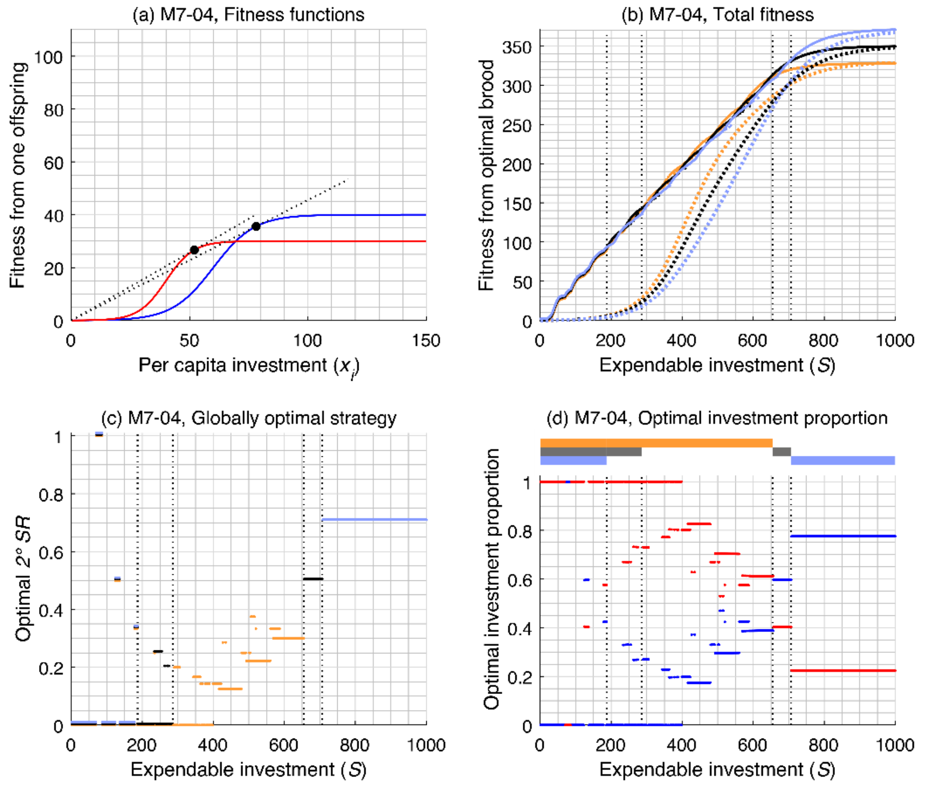

Figure S26. Fitness functions and corresponding globally optimal strategies of model *M7-4*. Panels are arranged as in Fig. S19. For more explanations, see Fig. S18 and caption to Fig. S19.

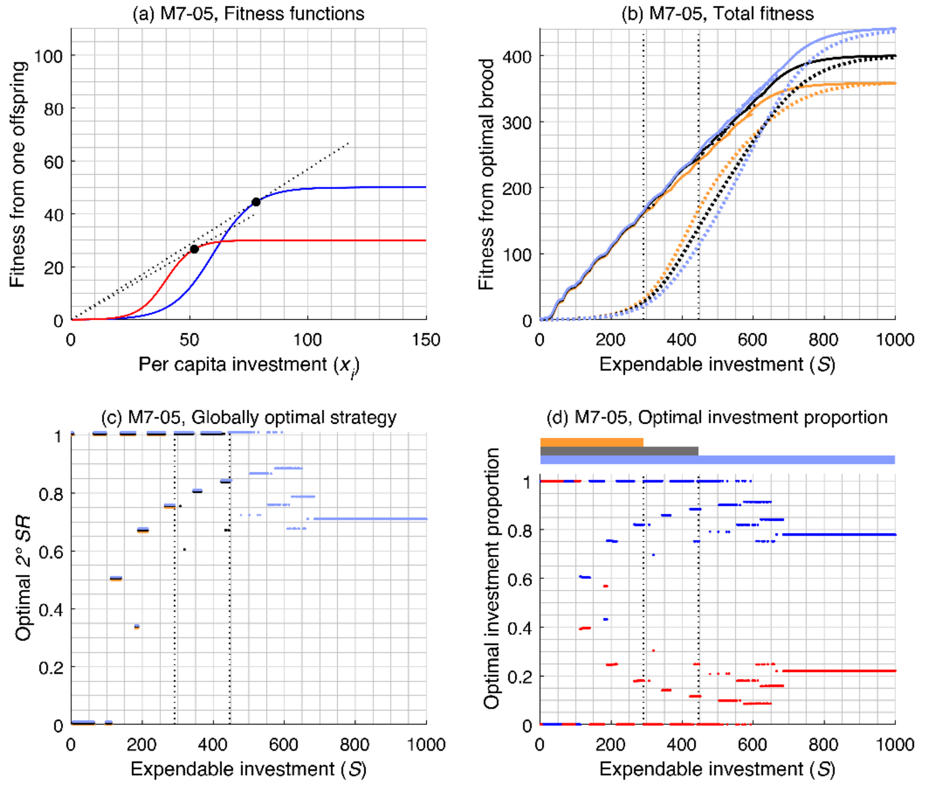

Figure S27. Fitness functions and corresponding globally optimal strategies of model *M7-5*. Panels are arranged as in Fig. S19. For more explanations, see Fig. S18 and caption to Fig. S19.

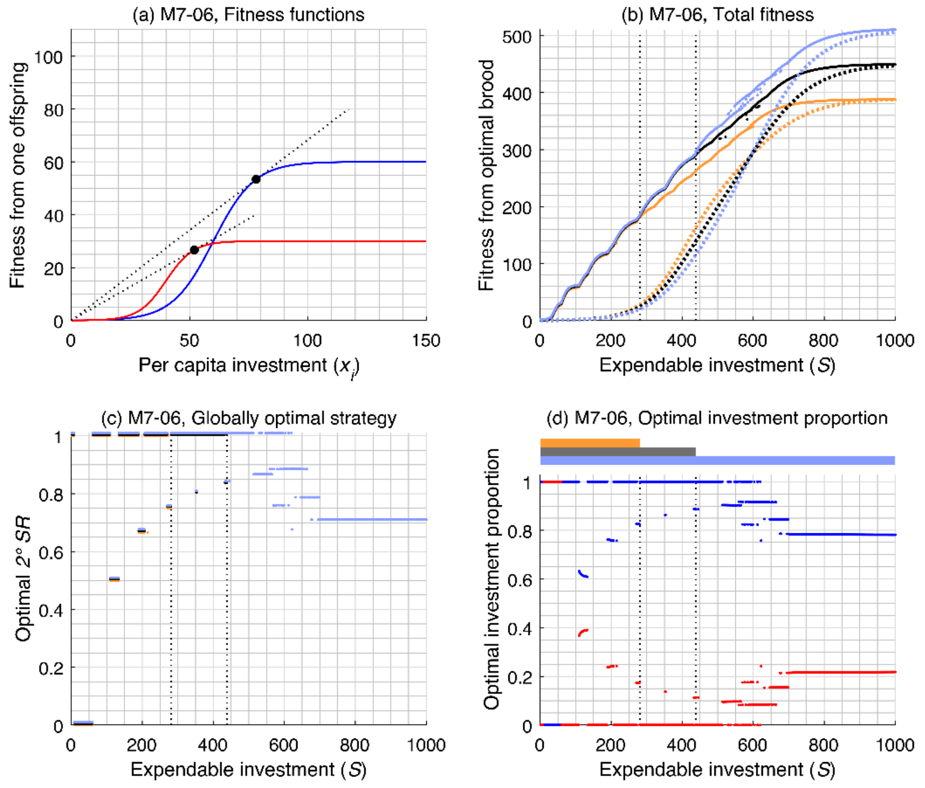

Figure S28. Fitness functions and corresponding globally optimal strategies of model *M7-6*. Panels are arranged as in Fig. S19. For more explanations, see Fig. S18 and caption to Fig. S19.

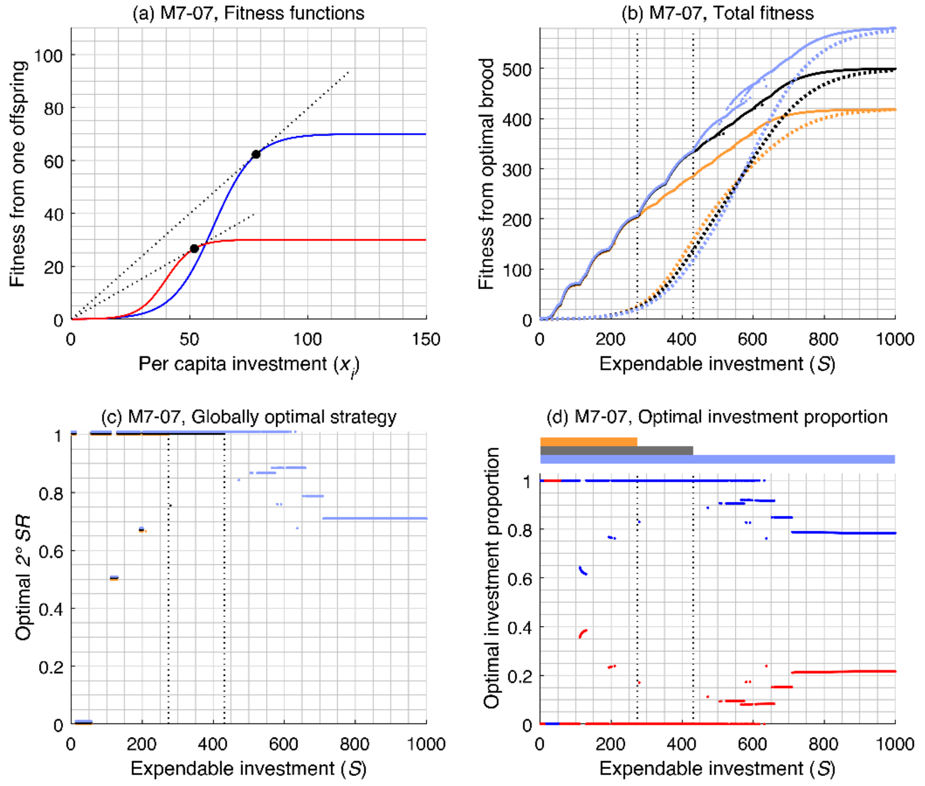

Figure S29. Fitness functions and corresponding globally optimal strategies of model *M7-7*. Panels are arranged as in Fig. S19. For more explanations see Fig. S18 and caption to Fig. S19.

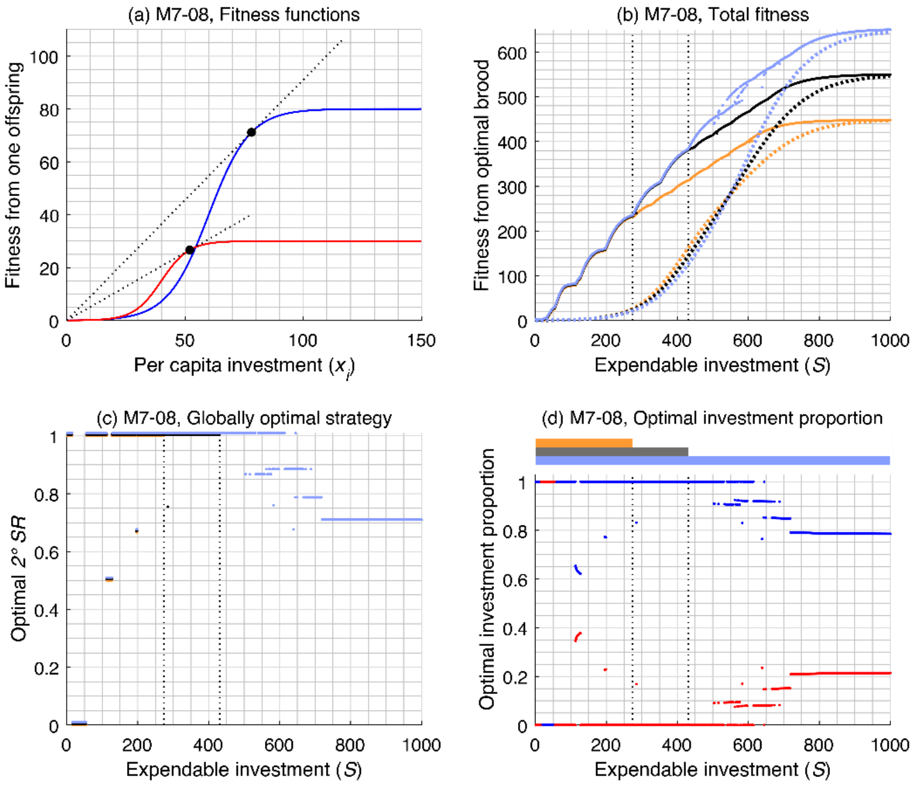

Figure S30. Fitness functions and corresponding globally optimal strategies of model *M7-8*. Panels are arranged as in Fig. S19. For more explanations see Fig. S18 and caption to Fig. S19.

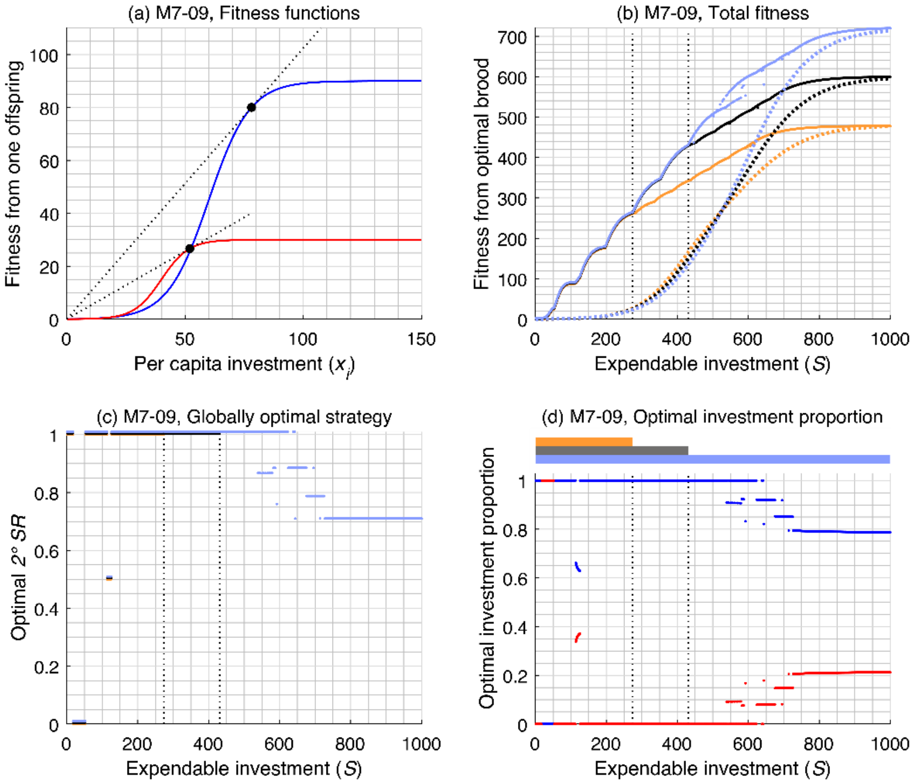

Figure S31. Fitness functions and corresponding globally optimal strategies of model *M7-9*. Panels are arranged as in Fig. S19. For more explanations see Fig. S18 and caption to Fig. S19.

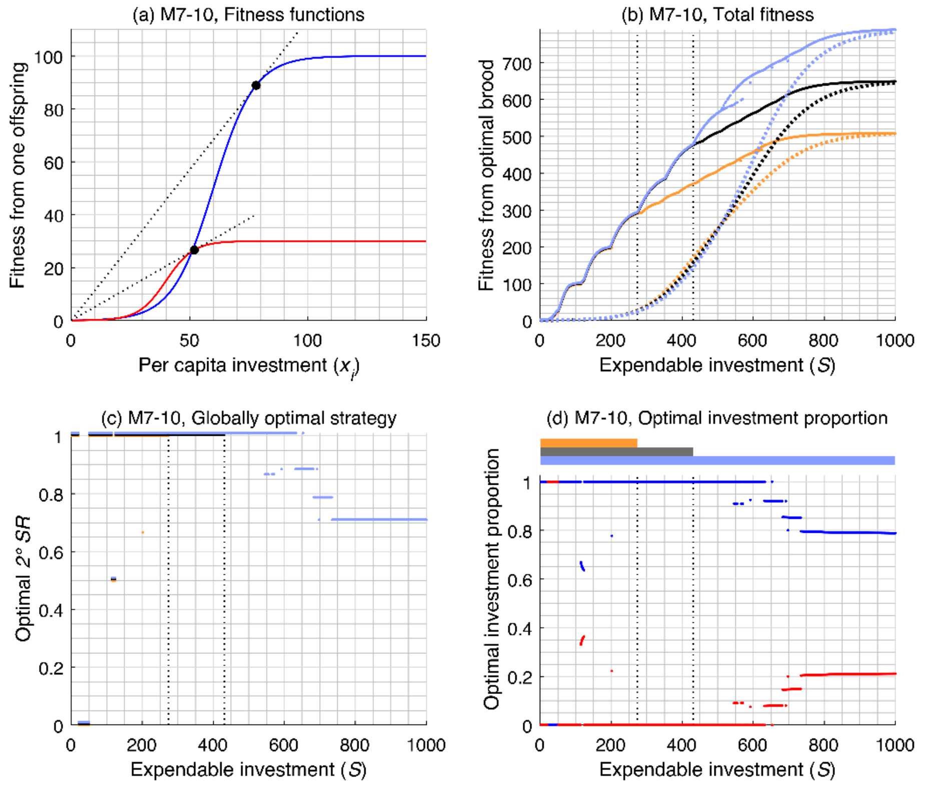

Figure S32. Fitness functions and the corresponding globally optimal strategies of model *M7-10*. Panels are arranged as in Fig. S19. For more explanations see Fig. S18 and caption to Fig. S19.

Table S3. Characteristics of globally optimal strategies assuming that an organism is able to use the best combination of *1° SR* (from the three ratios: 7:3, 5:5, 3:7) and investment rules, compared to the classical TWH predictions. Global optimal strategies contain locally optimal strategies from Table S2 for the fitness-maximizing *1° SRs.* Predictions from the classical TWH (column 5) are solely based on the variance (range) of each fitness function. The change of bias as *S* increases is a crucial element of predictions from the classical TWH. *Sex*-*1*-biased means that *Sex 1* comprises >50% of brood or that *Sex 1* receives >50% of total investment (*S*). The table concerns panels of Fig. S19-S32 that contain information pertinent to the specific columns (columns 2, 3, 4) as explained in the table's header. Trends in model results that are compared to the TWH predictions concern broods with multiple offspring, ignoring the patterns for very small *S* when optimal brood is composed of one offspring.

	**Model results**			**Prediction from classical TWH**	**General/approximate conformity between the classical TWH predictions and the model results**		
**1**	**2**	**3**	**4**	**5**	**6**	**7**	**8**
Model name/ figure number	The trend of globally optimal *1° SR* as *S* increases (panel (c), (d))	The general tendency of globally optimal *2° SR* as *S* increases (panel (c))	The general tendency of globally optimal allocation of investment between sexes as *S* increases (panel (d))	Sex that should be favored under TWH with high *S*	Optimal *1° SR*	Optimal *2° SR*	Optimal allocation of *S* between the two sexes
M3/ S19	*Sex*-*2*-biased	Broods become *Sex*-*2*-biased	*Sex*-*2*-biased	*Sex 2*	Consistent	Consistent	Consistent
M4/ S20	*Sex*-*2*-biased	Broods become *Sex*-*2*-biased	*Sex*-*2*-biased	*Sex 2*	Consistent	Consistent	Consistent
M5/ S21	*Sex*-*2*-biased	Initial scatter, then increase in *Sex*-*1* (but not *Sex*-*1*-biased)	Initial scatter, then increase in *Sex 1* (but not *Sex*-*1*-biased)	*Sex 2*	Consistent	Inconsistent	Inconsistent
M6/ S22	*Sex*-*2*-biased	Initial scatter, then increase in *Sex*-*1* (but not *Sex*-*1*-biased)	Initial scatter, then increase in *Sex 1*, becoming *Sex*-*1*-biased	No favored sex	Inconsistent	Inconsistent	Inconsistent
M7-1/ S23	*Sex*-*1*-biased	Increase in *Sex*-*2* (but not *Sex*-*2*-biased)	Increase in *Sex*-*2* (but not *Sex*-*2*-biased)	*Sex 1*	Consistent	Inconsistent	Inconsistent
M7-2/ S24	*Sex*-*1*-biased	Increase in *Sex*-*2* (but not *Sex*-*2*-biased)	Increase in *Sex*-*2* (but not *Sex*-*2*-biased)	*Sex 1*	Consistent	Inconsistent	Inconsistent
M7-3/ S25	*Sex*-*1*-biased	Increase in *Sex*-*2* (but not *Sex*-*2*-biased)	Increase in *Sex*-*2* (but not *Sex*-*2*-biased)	No favored sex	Inconsistent	Inconsistent	Inconsistent
M7-4/ S26	*Sex*-*2*-biased	Initial scatter, then increase in *Sex*-*2*, becoming *Sex*-*2*-biased	Initial scatter, then increase in *Sex 2*, becoming *Sex*-*1*-biased	*Sex 2*	Consistent	Consistent	Consistent
M7-5/ S27	*Sex*-*2*-biased	Initial scatter, then increase in *Sex 1* (but not *Sex*-*1*-biased)	Initial scatter, then increase in *Sex 1* (but not *Sex*-*1*-biased)	*Sex 2*	Consistent	Inconsistent	Inconsistent
M7-6/ S28	*Sex*-*2*-biased	Initial scatter, then increase in *Sex 1* (but not *Sex*-*1*-biased)	Initial scatter, then increase in *Sex 1* (but not *Sex*-*1*-biased)	*Sex 2*	Consistent	Inconsistent	Inconsistent
M7-7/ S29	*Sex*-*2*-biased	Initial scatter, then increase in *Sex 1* (but not *Sex*-*1*-biased)	Initial scatter, then increase in *Sex 1* (but not *Sex*-*1*-biased)	*Sex 2*	Consistent	Inconsistent	Inconsistent
M7-8/ S30	*Sex*-*2*-biased	Initial scatter, then increase in *Sex 1* (but not *Sex*-*1*-biased)	Initial scatter, then increase in *Sex*-*1* (but not *Sex*-*1*-biased)	*Sex 2*	Consistent	Inconsistent	Inconsistent
M7-9/ S31	*Sex*-*2*-biased	Initial scatter, then increase in *Sex 1* (but not *Sex*-*1*-biased)	Initial scatter, then increase in *Sex*-*1* (but not *Sex*-*1*-biased)	*Sex 2*	Consistent	Inconsistent	Inconsistent
M7-10/ S32	*Sex*-*2*-biased	Increase in *Sex 1* (but not *Sex*-*1*-biased)	Initial scatter, then increase in *Sex*-*1* (but not *Sex*-*1*-biased)	*Sex 2*	Consistent	Inconsistent	Inconsistent

Table S4. Comparison of fitness function properties and observed investment patterns of optimal parental strategies. Slope of the tangent through the origin predicts the general pattern of optimal allocation (expressed as changes in the relative proportion of S invested into each sex in response to S). n^* refers to the optimal number of cared offspring, regardless of *Sex 1* or *Sex 2*, out of 10 offspring. Predictions from classical TWH are solely based on difference in variance/range between fitness functions.

	**Properties of *Sex 1* and *Sex 2* functions**							**The general pattern of locally optimal strategy**			**The general pattern of globally optimal strategy**	
Model name/ figure number	Sharp increase in steepness with smaller *S* (at the inflection point)	Attainment of asymptote with smaller *S*	Greater *per capita* investment with higher *S* (n*=10, but *S*≤1000)	Female in classical TWH: attainment of maximum with smaller *S* and lower asymptote	Male in classical TWH: attainment of maximum with larger *S* and higher asymptote	Higher asymptote	The higher slope of the tangent line through the origin	Sex generally invested in when *S* is relatively low (but n*≥2)	The general direction of changes in the proportion of *S* with increasing *S* for *Sex 1*	Greater investment with high *S* (n*=10, but *S*≤1000)	Sex generally invested in when *S* is relatively low (but n*≥2): investment is inversely proportional to *S* in this sex (panel (d) in Fig. S19-S32)	The approximate trend in the 2° *SR*: the sex prevalent in broods of parents with small *S* (but n*≥2, panel (c) in Fig. S19-S32)
M3/ S3	*Sex 1*	*Sex 1*	*Sex 2*	*Sex 1*	*Sex 2*	*Sex 2*	*Sex 1*	*Sex 1*	Decrease	*Sex 2*	*Sex 1*	*Sex 1*
M4/ S4	*Sex 1*	*Sex 1*	*Sex 2*	*Sex 1*	*Sex 2*	*Sex 2*	*Sex 1*	*Sex 1*	Decrease	*Sex 2*	*Sex 1*	*Sex 1*
M5/ S5	*Sex 1*	*Sex 2*	*Sex 1*	*Sex 1*	*Sex 2*	*Sex 2*	*Sex 2*	*Sex 2*	Increase	*Sex 1* in 7:3 and 5:5; but *Sex 2* in 3:7 broods	*Sex 2*	*Sex 2*
M6/ S6	Equal	*Sex 2*	*Sex 1*	Discordance with the categorization of classical TWH		Equal	*Sex 2*	*Sex 2*	Increase	*Sex 1*	*Sex 2*	*Sex 2*
M7-1/ S7	*Sex 1*	*Sex 1*	*Sex 2*	Discordance with the categorization of classical TWH		*Sex 1*	*Sex 1*	*Sex 1*	Decrease	*Sex 1* in 7:3; but *Sex 2* in 3:7 and 5:5 broods	*Sex 1*	*Sex 1*
M7-2/ S8	*Sex 1*	*Sex 1*	*Sex 2*	Discordance with the categorization of classical TWH		*Sex 1*	*Sex 1*	*Sex 1*	Decrease	*Sex 1* in 7:3; but *Sex 2* in 3:7 and 5:5 broods	*Sex 1*	*Sex 1*
M7-3/ S9	*Sex 1*	*Sex 1*	*Sex 2*	Discordance with the categorization of classical TWH		Equal	*Sex 1*	*Sex 1*	Decrease	*Sex 1* in 7:3; but *Sex 2* in 3:7 and 5:5 broods	*Sex 1*	*Sex 1*
M7-4/ S10	*Sex 1*	*Sex 1*	*Sex 2*	*Sex 1*	*Sex 2*	*Sex 2*	*Sex 1*	*Sex 1*	Decrease	*Sex 1* in 7:3; but *Sex 2* in 3:7 and 5:5 broods	*Sex 1*	*Sex 1*
M7-5/ S11	*Sex 1*	*Sex 1*	*Sex 2*	*Sex 1*	*Sex 2*	*Sex 2*	*Sex 2*	*Sex 2*	Increase	*Sex 1* in 7:3; but *Sex 2* in 3:7 and 5:5 broods	*Sex 2*	*Sex 2*
M7-6/ S12	*Sex 1*	*Sex 1*	*Sex 2*	*Sex 1*	*Sex 2*	*Sex 2*	*Sex 2*	*Sex 2*	Increase	*Sex 1* in 7:3; but *Sex 2* in 3:7 and 5:5 broods	*Sex 2*	*Sex 2*
M7-7/ S13	*Sex 1*	*Sex 1*	*Sex 2*	*Sex 1*	*Sex 2*	*Sex 2*	*Sex 2*	*Sex 2*	Increase	*Sex 1* in 7:3; but *Sex 2* in 3:7 and 5:5 broods	*Sex 2*	*Sex 2*
M7-8/ S14	*Sex 1*	*Sex 1*	*Sex 2*	*Sex 1*	*Sex 2*	*Sex 2*	*Sex 2*	*Sex 2*	Increase	*Sex 1* in 7:3; but *Sex 2* in 3:7 and 5:5 broods	*Sex 2*	*Sex 2*
M7-9/ S15	*Sex 1*	*Sex 1*	*Sex 2*	*Sex 1*	*Sex 2*	*Sex 2*	*Sex 2*	*Sex 2*	Increase	*Sex 1* in 7:3; but *Sex 2* in 3:7 and 5:5 broods	*Sex 2*	*Sex 2*
M7-10/ S16	*Sex 1*	*Sex 1*	*Sex 2*	*Sex 1*	*Sex 2*	*Sex 2*	*Sex 2*	*Sex 2*	Increase	*Sex 1* in 7:3; but *Sex 2* in 3:7 and 5:5 broods	*Sex 2*	*Sex 2*

Part 4 Mathematical theorems and proofs

General Assumptions

Fitness functions of individuals, $f_i(x)$ and $f_j(x)$ are logistic which are continuous and differentiable on $x \in \mathbf{R}$. The conditions of fitness functions include,

(1) The fitness functions have positive inflection points,

(2) zero if and only if $x = 0$.

In our study, fitness functions are expressed as

$$f_i(x) = \frac{\alpha_i}{1 + e^{-\beta_i(x-\gamma_i)}} + \delta_i$$

where α_i, β_i, δ_i and γ_i are coefficients.

X is defined as $X = (x_1, x_2, x_3, \dots, x_N)$ (where $0 \le x_i \le S$ and $\sum x_i = S$). Let $X^* = (v_1, v_2, v_3, \dots, v_N)$ be the solution of the optimization problem where $F(X) = \sum_{i=1}^{N} f_{b_i}(x_i)$, then for any $X \neq X^*$, $F(X) \le F(X^*)$ holds. As proven in subsequent propositions, X^* may not be unique. Take F^* as a function of S such that $F^*(S) = F(X^*) = \sum_{i=1}^{N} f_i(v_i)$.

Lemma 1

Let $X^* = (v_1, v_2, v_3, \dots, v_N)$ be the solution of the optimization problem, then $f'_i(v_i) = f'_j(v_j)$ for any $v_i,\ v_j > 0\ (i \neq j)$.

Proof

Karush-Kuhn-Tucker conditions were used to prove this **Lemma**. First, we write the optimization problem as

Maximize $F(X) = f_1(x_1) + f_2(x_2) + f_3(x_3) + \cdots + f_N(x_N)$

subject to $\begin{cases} x_1, x_2, x_3, \cdots, x_N \ge 0 \\ x_1 + x_2 + x_3 + \cdots + x_N - S = 0 \end{cases}$

Note that we can rewrite the constraints as

$$\begin{cases} g_i(X) = -x_i \le 0 \quad \text{for } i = 1,2,3,\cdots,N \\ h(X) = x_1 + x_2 + x_3 + \cdots + x_N - S = 0. \end{cases}$$

Here, one can form the Lagrange function $L(X, \mu, \lambda) = F(X) + \sum_{i=1}^{N} \mu_i g_i(X) + \lambda h(X)$ where $\mu = (\mu_1, \mu_2, \mu_3, \cdots, \mu_N)$.

By the **KKT theorem**, for any solution $X^* = (v_1, v_2, v_3, \dots, v_N)$ of optimization problem, there exist $\mu_1, \mu_2, \mu_3, \cdots, \mu_N$ and λ satisfying

$$\begin{cases} \nabla F(X^*) - \sum_{i=1}^{N} \mu_i \nabla g_i(X^*) - \lambda \nabla h(X^*) = 0 \\ \mu_i g_i(X^*) = 0 \quad \text{for } i = 1,2,3,\cdots,N \end{cases}$$

and hence

$$\begin{cases} f_i'(v_i) + \mu_i - \lambda = 0 \\ \mu_i v_i = 0 \end{cases} \qquad \text{for } i = 1,2,3,\cdots,N$$

Now consider any $v_i,\ v_j > 0$ in X^*. Since $v_i \neq 0$, it can be expected that $\mu_i = 0$ and $f_i'(v_i) = \lambda$. Similarly, one can get $f_j'(v_j) = \lambda$ and therefore $f_i'(v_i) = f_j'(v_j)$.

Definition 1

Selective equitable distribution (henceforth 'SED') is a strategy of parental care distribution by which one selects n offspring and provides equitable care to those n offspring ($1 \leq n \leq N$). In other words, X is SED if X is composed of a (> 0) or 0.

Definition 2

Let p^* be the inflection point of the fitness function. Let N_L be the number of components in X^* that are less than p^* (not including 0). Similarly, let N_H be the number of components in X^* that are larger than p^*. Let β_i's ($\beta_i > 0$) be the components in X^* which are less than p^*, and let γ_i's be the components in X^* which are greater than p^*. In other words, $\mathrm{B} = \{\beta_1, \beta_2, \beta_3, \cdots, \beta_{N_L}\}$ is a set of the nonzero x-values of the optimal point that are less than p^*. $\Gamma = \{\gamma_1, \gamma_2, \gamma_3, \cdots, \gamma_{N_H}\}$, on the other hand, is a set of the nonzero x-values of the optimal point that are larger than p^*.

Remark 1

Assume that all fitness functions are identical. By **Lemma 1**, $f'(v_i)$ should be the same for every $v_i > 0$. For v_i and v_j to differ while $f'(v_i) = f'(v_j)$, they should be symmetric to the inflection point p^* as f' is a symmetric unimodal function. By unimodality, $f'(x)$ monotonically increases and thus injective for $x < p^*$. As $f'(\beta_i) = f'(\beta_j)$ should hold, $\beta_i = \beta_j$. Similarly, $\gamma_i = \gamma_j$. Take $\beta = \beta_i$ and $\gamma = \gamma_i$, then $\frac{(\beta+\gamma)}{2} = p^*$ should be satisfied due to the symmetry of f'.

Theorem 1 (Identical derivative in optimal distribution)

When all fitness functions are identical (say f), the solution of optimization problem is SED given that $S \neq 2p^*$.

Proof

Take an optimal solution of the optimization problem, $X^* = (v_1, v_2, v_3, \dots, v_N)$, and consider the following cases.

(i) $N_L = 0$ and $N_H = 0$

X^* has no nonzero components that are less or larger than p^* which means each v_i is either 0 or p^*. Therefore X^* is SED.

(ii) $N_L = 0$ and $N_H \geq 1$

$X^* = (\gamma_1, \gamma_2, \gamma_3, \cdots, \gamma_{N_H}, 0, \cdots, 0)$ since $N_L = 0$. No component of X^* is equal to p^* by **Lemma 1**

because $f'(\gamma_1) \neq f'(p^*)$. Since $\gamma_1 = \gamma_2 = \gamma_3 = \cdots = \gamma_{N_H} = \gamma$ by **Remark 1**, X^* is SED.

(iii) $N_L \geq 1$ and $N_H = 0$

Similar to (ii), X^* is SED.

(iv) $N_L \geq 2$ and $N_H \geq 1$

β_1, β_2 and γ_1 are the components of $X^* = (v_1, v_2, v_3, \ldots, v_N)$. Without loss of generality, one can assign $v_1 = \gamma_1$, $v_2 = \beta_1$, and $v_3 = \beta_2$. Then $\beta_1 = \beta_2 = \beta$ and $\gamma_1 = \gamma$. By symmetry of f', $f'(\beta_1) = f'(\gamma_1)$ implies that $p^* - \beta = \gamma - p^*$. Since f is point symmetry to the inflection point, we have $f(\beta_1) + f(\gamma_1) = f(p^*) + f(p^*)$. Therefore $F(X^*) = \sum_{i=1}^{N} f(v_i) = \sum_{i=3}^{N} f(v_i) + 2f(p^*)$, and thus $\tilde{X} = (p^*, p^*, \beta, v_4, \ldots, v_N)$ is also a solution of the optimization problem. The contraposition of **Lemma 1** indicates that $\tilde{X} = (p^*, p^*, \beta, v_4, \ldots, v_N)$ cannot be optimal since $f'(p^*) \neq f'(\beta)$ which is contradictory.

(v) $N_L \geq 1$ and $N_H \geq 2$

Similarly, this condition cannot hold because of **Lemma 1**.

(vi) $N_L = 1$ and $N_H = 1$

$f(\beta_1) + f(\gamma_1) = f(p^*) + f(p^*)$ should hold by symmetricity of f' and f. Due to **Lemma 1**, every component of X^* other than β_1 or γ_1 should be 0 as $f'(p^*) \neq f'(\beta_1) = f'(\gamma_1)$. Thus $F(X^*) = \sum_{i=1}^{N} f(v_i) = f(\beta_1) + f(\gamma_1) = f(p^*) + f(p^*)$. Therefore, if $N_L = 1$ and $N_H = 1$ hold, then $S = 2p^*$ should be satisfied.

Corollary of Theorem 1

The solution of the optimization problem is not necessarily unique. When a distribution solution X^* is optimal and is not SED, $N_L = N_H = 1$ and $\frac{(\beta+\gamma)}{2} = p^*$ should be satisfied. In this case, $X = (p^*, p^*, 0,0,0, \ldots, 0)$, which is SED, is also a solution of optimization problem.

Proof

From the proof of **Theorem 1**, one can get $N_L = N_H = 1$ if X^* is not SED. By symmetry of f', $f'(\beta_1) = f'(\gamma_1)$ gives us that $p^* - \beta_1 = \gamma_1 - p^*$, or $\frac{(\beta_1+\gamma_1)}{2} = p^*$.

Now, to show that X^* is not a unique optimal strategy, let us take $0 < \varepsilon < (p^* - \beta_1)$ and $0 < \omega < \beta_1$. Again, by symmetry,

$$\int_{\beta_1}^{\beta_1+\varepsilon} f'(t)dt = \int_{\gamma_1-\varepsilon}^{\gamma_1} f'(t)dt$$

and

$$\int_{\beta_1-\omega}^{\beta_1} f'(t)dt = \int_{\gamma_1}^{\gamma_1+\omega} f'(t)dt.$$

This produces that,

$$f(\beta+\varepsilon)+f(\gamma-\varepsilon)=f(\beta)+\int_{\beta}^{\beta+\varepsilon} f'(t)dt+f(\gamma)+\int_{\gamma}^{\gamma-\varepsilon} f'(t)dt \ =f(\beta)+f(\gamma)$$

and similarly,

$$f(\beta-\omega)+f(\gamma+\omega)=f(\beta)+f(\gamma).$$

Therefore, the optimal solution is not fixed, but multiple optima are derived. This condition, however, is narrow because $N_L=N_H=1$ and $\frac{(\beta_1+\gamma_1)}{2}=p^*$ (and hence $S=2p^*$) should be satisfied. Otherwise, v_i's are the same ($v_i>0$) for the group of identical fitness functions.

Definition 2

Let ψ be the function defined by $\psi(x)=\frac{f(x)}{x}$ who has the maximum point at $x=a^*$.

Theorem 2 (Optimal distribution for identical fitness functions)

For a brood composed of offspring with identical logistic fitness functions, the number of the offspring who are cared of can be represented as a summation of floor functions where $S\neq S_n$.

Proof

According to **Corollary of Theorem 1**, SED is a solution of the optimization problem. As SED is optimal, one needs to find $n^*\in\{1,2,3,\cdots,N\}$ that maximizes $nf\left(\frac{S}{n}\right)$.

We use the function $\psi(x)=\frac{f(x)}{x}$. As $\psi(x)$ is a unimodal function that has a peak at $x=a^*$, ψ satisfies following conditions

$$\psi\left(\frac{S}{n}\right)>\psi\left(\frac{S}{n+1}\right)\ \text{ if }\ \frac{S}{n}<a^*$$
$$\psi\left(\frac{S}{n}\right)<\psi\left(\frac{S}{n+1}\right)\ \text{ if }\ \frac{S}{n+1}>a^*.$$

We can rewrite our problem as finding n^* satisfying

$$n^*=\underset{n}{\operatorname{argmax}}\ nf\left(\frac{S}{n}\right)=\ \underset{n}{\operatorname{argmax}}\ \frac{f\left(\frac{S}{n}\right)}{\frac{1}{n}}$$
$$=\ \underset{n}{\operatorname{argmax}}\ S\left(\frac{f\left(\frac{S}{n}\right)}{\frac{S}{n}}\right)=\ \underset{n}{\operatorname{argmax}}\ \psi\left(\frac{S}{n}\right)$$

where S is given. Let us divide S into three cases.

(i) $S<a^*$

$n^* = 1$ because $f(S) > nf\left(\frac{S}{n}\right)$ for $n \geq 2$ as $\psi(S) > \psi\left(\frac{S}{n}\right)$.

(ii) $a^* \leq S < Na^*$

Take $k \in \{1,2,3,\dots,N\}$ as the integer which satisfies the inequality condition $\frac{S}{k+1} < a^* \leq \frac{S}{k}$. Here, n^* is either k or $k+1$ because $\psi(x)$ has its peak at $x = a^*$. In case of $\frac{S}{k} = a^*$, $n^* = k$. As $k \leq \frac{S}{a^*} < k+1$, so that $k = \left[\frac{S}{a^*}\right]$ where [] is a floor function.

(iii) $Na^* \leq S$

$n^* = N$ because $Nf\left(\frac{S}{N}\right) > nf\left(\frac{S}{n}\right)$ for $n < N$, as $\psi\left(\frac{S}{N}\right) > \psi\left(\frac{S}{n}\right)$.

To sum up,

$n^* = 1$	$(S < a^*)$
$n^* = k$ or $n^* = k+1$	$(ka^* \leq S < (k+1)a^*,\ k \in \{1,2,3,\dots,(N-1)\})$
$n^* = N$	$(S \geq Na^*)$

Consider a function Ψ defined by $\Psi(S) = \psi\left(\frac{S}{k+1}\right) - \psi\left(\frac{S}{k}\right)$ where $k = \left[\frac{S}{a^*}\right]$. When take S such that $k = \frac{S}{a^*}$, $\Psi(S)$ becomes smaller than zero because $\psi\left(\frac{ka^*}{k+1}\right) < \psi\left(\frac{ka^*}{k}\right)$. Meanwhile,

$$\begin{aligned}\lim_{h\to0^+}\Psi\big((k+1)a^* - h\big) &= \lim_{h\to0^+}\left(\psi\left(\frac{(k+1)a^* - h}{k+1}\right) - \psi\left(\frac{(k+1)a^* - h}{k}\right)\right)\\ &= \lim_{h\to0^+}\psi\left(\frac{(k+1)a^* - h}{k+1}\right) - \lim_{h\to0^+}\psi\left(\frac{(k+1)a^* - h}{k}\right)\\ &= \lim_{h\to0^+}\psi\left(a^* - \frac{h}{k+1}\right) - \lim_{h\to0^+}\psi\left(a^* + \frac{a^*}{k} - \frac{h}{k}\right)\\ &> 0\end{aligned}$$

and hence, there is some $\varepsilon > 0$ satisfying $\Psi\big((k+1)a^* - \varepsilon\big) > 0$ and $ka^* < (k+1)a^* - \varepsilon$.

As ψ is continuous and differentiable, $\Psi(S)$ is accordingly continuous and differentiable in $ka^* \leq S < (k+1)a^*$. In addition, Ψ is (strictly) monotonically increasing because $\psi\left(\frac{S}{k+1}\right)$ is (strictly) monotonically increasing, and $\psi\left(\frac{S}{k}\right)$ is (strictly) monotonically decreasing in $ka^* \leq S < (k+1)a^*$. Due to **Intermediate Value Theorem**, there is $S_k \in (ka^*, (k+1)a^*)$ such that $\Psi(S) > 0$ if $S > S_k$ and $\Psi(S) < 0$ if $S < S_k$ (note that S_k is unique because Ψ is a monotonically increasing function within the range), which gives us

$$n^* = \begin{cases} k & \text{if} \quad ka^* \leq S < S_k \\ k+1 & \text{if} \quad S_k < S \leq (k+1)a^*. \end{cases}$$

where $k \in \{1,2,3,\dots,(N-1)\}$ and $S \neq S_k$. Note that both $n^* = k$ and $n^* = k+1$ are solutions of optimization problem if $S = S_k$. Therefore, $(k+1)\ f\left(\frac{S_k}{k+1}\right) = k\ f\left(\frac{S_k}{k}\right)$.

Finally, let A be the set of all S_k's, and let $\tau_{S_k}(x)$ be the step function such that

$$\tau_{S_k}(x) = \begin{cases} 0 & \text{if } x \leq S_k \\ 1 & \text{if } x > S_k. \end{cases}$$

Let $n^*(S)$ represent the optimized number of cared offspring by S. Then $n^*(S)$ can be represented by the sum of those step functions

$$n^*(S) = 1 + \sum_{S_k \in A} \tau_{S_k}(S).$$

Additionally, for a brood composed of offspring with identical logistic fitness functions, the optimal number of the cared offspring is not unique where $S \in A$. This is because both $n^*(S) = k$ and $n^*(S) = k + 1$ are solutions of optimization problem where $S = S_k \in A$.

Remark 2

For brevity let us write n^* instead of $n^*(S)$. Note that n^* is a function of S, which means that the value of S determines the value of n^*.

Definition 3

Define L_k and H_k as $\frac{S_k}{k+1}$ and $\frac{S_k}{k}$, respectively, such that $(k+1)\ f\left(\frac{S_k}{k+1}\right) = k\ f\left(\frac{S_k}{k}\right)$ holds.

Lemma 2

L_k and H_k are uniquely determined by the identical fitness function f. Especially, $L_1 = p^*$ and $H_1 = 2p^*$.

Proof

From the proof of **Theorem 2**, we know that there exists a unique S_k satisfying $(k+1)\ f\left(\frac{S_k}{k+1}\right) = k\ f\left(\frac{S_k}{k}\right)$. Therefore L_k and H_k are unique.

Now let us assume that we have $k = 1$. Figure S33 shows that the point symmetry of f gives us $\frac{f(p^*)}{p^*} = \frac{f(2p^*)}{2p^*}$ and hence $2f(p^*) = f(2p^*)$. Here one can easily get $S_1 = 2p^*$ because $2f\left(\frac{S_1}{2}\right) = 2f(p^*)$ and $f(S_1) = f(2p^*)$.

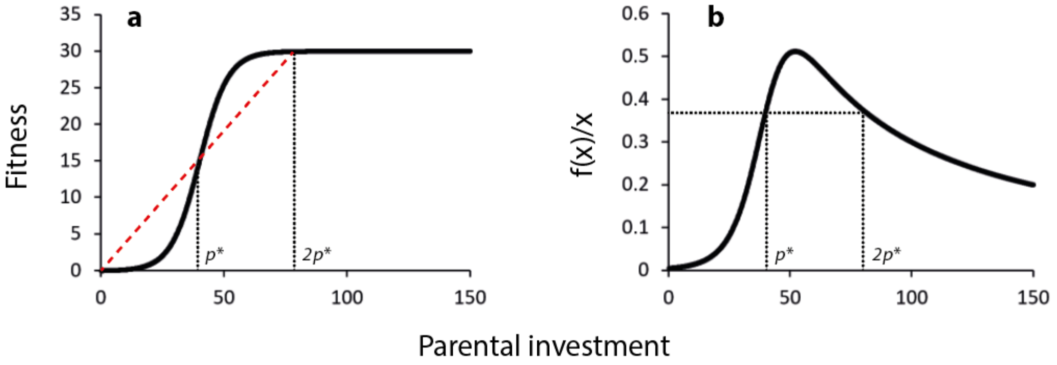

Figure S33. The graphical illustration of p^*.

(a) For a logistic fitness function, $(p^*, f(p^*))$ is an inflection point. Due to the point symmetry of logistic functions, $\frac{f(p^*)}{p^*} = \frac{f(2p^*)}{2p^*}$ holds.

(b) By the aforementioned symmetry, $\psi(p^*) = \psi(2p^*)$ holds where $\psi(x) = f(x)/x$.

Definition 4

Suppose that the brood is composed of two groups, each with a fitness function of f_1 or f_2. Then define S_1^* and S_2^* as a parental investment to each group that maximizes total fitness with the constraint that $S_1^* + S_2^* = S$. That is, $F_1(S_1^*) + F_2(S_2^*) = F^*(S)$ where $F_i(X)$ is the optimal summation of offspring fitness from i-th group to which X amount of care is given. Also, define $F(S_1, S_2) = F_1(S_1) + F_2(S_2)$ so that $F^*(S) = F(S_1^*, S_2^*) = F_1(S_1^*) + F_2(S_2^*)$.

Lemma 3

$F_1(X) = n_1^* f_1\left(\frac{X}{n_1^*}\right)$, $F_2(X) = n_2^* f_2\left(\frac{X}{n_2^*}\right)$ so that $F^*(S) = n_1^* f_1\left(\frac{S_1^*}{n_1^*}\right) + n_2^* f_2\left(\frac{S_2^*}{n_2^*}\right)$ where $S_2^* = S - S_1^*$.

Proof

For a given S_1^*, by **Corollary of Theorem 1**, $n_1^* f_1\left(\frac{S_1^*}{n_1^*}\right)$ is the optimal total fitness from group 1. Similarly, $n_2^* f_1\left(\frac{S_2^*}{n_2^*}\right)$ is the optimal total fitness from group 2. As total optimal fitness is the sum of total fitness from group 1 and 2, $F^*(S) = F_1(S_1^*) + F_2(S_2^*) = n_1^* f_1\left(\frac{S_1^*}{n_1^*}\right) + n_2^* f_2\left(\frac{S_2^*}{n_2^*}\right)$. By the constraint condition that $S_1 + S_2 = S$, it follows that $S_2^* = S - S_1^*$.

Algorithm 1: Based on **Theorem 2**, the optimal number of the cared offspring for i-th group is

$$n_i^* = \underset{n_i \in \{k, k+1\}}{\operatorname{argmax}} \; n_i f_i\left(\frac{S_i}{n_i}\right)$$

where $k = \left\lfloor \frac{S}{a^*} \right\rfloor$. Therefore, by **Lemma 3**,

$$F^*(S) = n_1^* f_1\left(\frac{S_1^*}{n_1^*}\right) + n_2^* f_2\left(\frac{S - S_1^*}{n_2^*}\right)$$

As $F(S)$ is a univariate function of S_1,

$$S_1^* = \underset{S_1}{\operatorname{argmax}}\left\{n_1^* f_1\left(\frac{S_1}{n_1^*}\right) + n_2^* f_2\left(\frac{S - S_1}{n_2^*}\right)\right\}$$

And

$$S_2^* = S - S_1^*.$$

Note that one could reduce the computational demands by the factor of $2/N$. Without **Algorithm 1**, one has to compute $nf\left(\frac{S}{n}\right)$ for $n = 1, \cdots, N$ to find the optimal n^*. But now thanks to the **Theorem 2**, one only needs to compute $nf\left(\frac{S}{n}\right)$ for $n = k$ and $n = k + 1$ where $k = \left[\frac{S}{a^*}\right]$.

Lemma 4

Suppose that all fitness functions are identical. If S is smaller than $a^* N$, then the non-zeros optimal parental care given to each offspring is $\frac{S}{n^*}$ which lies between L_{n^*-1} and H_{n^*}.

Proof

By **Theorem 1**, the solution X^* of the optimization problem is SED and therefore, the optimal parental care is either $\frac{S}{n^*}$ or zero.

According to proof of **Theorem 2**, the following holds.

$$n^* = \begin{cases} k & \text{if} \quad ka^* \leq S < S_k \\ k+1 & \text{if} \quad S_k < S \leq (k+1)a^* \end{cases}$$

where $k = [S/a^*]$. If $n^* = k$, then $S_{n^*-1} < n^* a^* \leq S < S_k = S_{n^*}$. On the other hand, if $n^* = k + 1$, then $S_{n^*-1} = S_k < S \leq n^* a^* < S_{n^*}$. Therefore, we have $S_{n^*-1} < S < S_{n^*}$ if $S \neq S_k$ (For simplicity, set $S_0 = 0$ and $L_0 = 0$).

By the definition, $H_{n^*} = \frac{S_{n^*}}{n^*}$ and $L_{n^*-1} = \frac{S_{n^*-1}}{n^*}$. Thus $L_{n^*-1} < \frac{S}{n^*} < H_{n^*}$ for $S \neq S_k$ and therefore $L_{n^*-1} \leq \frac{S}{n^*} \leq H_{n^*}$.

Theorem 3 (Optimal *per capita* investment)

Assume that N is sufficiently large (i.e., there are many offspring) and all fitness functions are identical. Then the non-zero optimal parental care given to each offspring $\frac{S}{n^*}$ converges to a^* as the total expendable care S grows large.

Proof

If $\psi(H_i) = \psi(L_i)$ and $L_i < H_i$, then $L_i < a^* < H_i$ due to unimodality of ψ. (Refer to Fig. S33b.)

Now define a function ϕ such that $\phi(K) = \frac{\alpha}{\beta}$ where α and β are the solutions of $\psi(x) = K$. Then ϕ is defined on $(\psi(0), \psi(a^*))$ and monotonically increasing. The range of ψ is (0,1).

Suppose $\phi(K) = \frac{n}{n+1}$ for some n. Then by the definition of ψ, $\frac{f(\beta)}{\beta} = \frac{f(\alpha)}{\alpha}$. As $\frac{\alpha}{\beta} = \frac{n}{n+1}$, both of $\alpha = \frac{S}{n+1}$ and $\beta = \frac{S}{n}$ for some S. As $\frac{f(S/(n+1))}{S/(n+1)} = \frac{f(S/n)}{S/n}$, by the definition of S_n, $S = S_n$. Thus $\alpha = L_n$ and $\beta = H_n$.

Suppose that $\phi(K_1) = \frac{n_1}{n_1+1} = \frac{\alpha_{n_1}}{\beta_{n_1}}$, $\phi(K_2) = \frac{n_2}{n_2+1} = \frac{\alpha_{n_2}}{\beta_{n_2}}$, and $n_1 < n_2$. As $\frac{n}{n+1}$ is a monotonically increasing sequence, $\phi(K_2) > \phi(K_1)$. As ϕ is monotonically increasing as well, $K_2 > K_1$. ψ monotonically increases when $0 < x < a^*$, and monotonically decreases when $a^* < x$. Therefore, $K_2 > K_1$ indicates that $\alpha_{n_1} < \alpha_{n_2}$ and $\beta_{n_1} > \beta_{n_2}$ due to the geometrical structure of ψ.

By Theorem 1, the solution X^* of the optimization problem is SED and therefore, the optimal parental care is either $\frac{S}{n^*}$ or zero. Since S grows large and N is sufficiently large, we can assume that $a^* < S \le Na^*$. We have to deal with two following cases.

(i) $S < Na^*$

According to **Lemma 4**, $\frac{S}{n^*}$ lies between L_{n^*-1} and H_{n^*}.

Here, $\frac{S}{n^*} \le H_{n^*}$ implies $\frac{S}{n^*+1} \le \frac{n^*}{n^*+1} H_{n^*} = \frac{S_{n^*}}{n^*+1} = L_{n^*}$ and $\frac{S}{n^*} \ge L_{n^*-1}$ gives us $\frac{S}{n^*-1} \ge \frac{n^*}{n^*-1} L_{n^*-1} = \frac{S_{n^*-1}}{n^*-1} = H_{n^*-1}$. Therefore,

$$\lim_{S\to\infty} H_{n^*-1} = \lim_{S\to\infty} H_{n^*} \le \lim_{S\to\infty} \frac{S}{n^*} \le \lim_{S\to\infty} L_{n^*}$$

where $\psi(H_n) = \psi(L_n)$. As $L_n \le a^* \le H_n$ for any n due to unimodality of the function ψ,

$$\lim_{S\to\infty} L_{n^*} \le a^* \le \lim_{S\to\infty} H_{n^*}$$

As L_n and H_n are monotonically increasing and decreasing, respectively, L_{n^*}, H_{n^*} and $\frac{S}{n^*}$ converge to a^* as S grows large.

(ii) $S = Na^*$

According to proof of **Theorem 2**, $n^* = N$ and therefore $\frac{S}{n^*} = a^*$.

Theorem 4 (Investment version of the TWH)

Suppose the following conditions hold.

(1) $f_1(a_1^*) > f_2(p_2^* + \frac{a_1^*}{2}) - f_2(p_2^* - \frac{a_1^*}{2})$.

(2) There is $L > 2p_1^*$ such that $f_1(x) > f_2(x)$ for $0 < x < L$, and $f_1(x) < f_2(x)$ for $x > L$.

(3) For *Sex 1*, the optimal number of offspring is smaller than the total number of offspring ($n_1^* < N_1$).

Then every investment goes to *Sex 1*. That is, $S_2^* = 0$ and $S_1^* = S$, which means $n_1^* > n_2^* = 0$.

Proof

Suppose that $S_2^* > 0$. As $n_1^* \leq \left[\frac{S}{a_1^*}\right] + 1 < N_1$, there is at least 1 *Sex 1* which does not receive the care. Define F_A the optimal total fitness in this condition.

(i) $v_2 = \frac{S_2^*}{n_2^*} < L$

Suppose that v_2 allocated to 1 *Sex 2* offspring is transferred to 1 *Sex 1* offspring which does not receive the care. As $f_1(v_2) > f_2(v_2)$, F_A is not optimal.

(ii) $v_2 = \frac{S_2^*}{n_2^*} \geq L$

Due to the condition (2), $v_2 \geq L > 2p_1^* > a_1^*$, therefore $v_2 - a_1^* > 0$. Suppose that the a_1^* amount of investment is transferred from 1 *Sex 2* offspring to which does not receive the care. As f_2' is symmetric unimodal and has a peak at $x = p_2^*$, it holds true that $f_2(v_2) - f_2(v_2 - a_1^*) \leq f_2\left(p_2^* + \frac{a_1^*}{2}\right) - f_2\left(p_2^* - \frac{a_1^*}{2}\right)$. By the condition (1), $f_2(v_2) - f_2(v_2 - a_1^*) < f_1(a_1^*)$. Thus F_A is not optimal.

(i) and (ii) indicate that F_A is not optimal which is a contradiction. By reduction to absurdity, $S_2^* = 0$.

Lemma 5.1

If $a_1^* < a_2^*$, $\frac{f_1(a_1^*)}{a_1^*} < 2\frac{f_2(a_2^*)}{a_2^*}$, and $S > a_2^*$, then $S_2^* > 0$ given that $N_2 \geq k_2$ where $ka_2^* \leq S < (k+1)a_2^*$.

Proof

Suppose $S_2^* = 0$ and $S_1^* = S$ hold. Let us denote the total fitness in this case as F_A. Then $F_A = F_1(S) = n_1^* f_1\left(\frac{S}{n_1^*}\right) = S\psi_1\left(\frac{S}{n_1^*}\right) \leq S\psi_1(a_1^*)$. As $k_2 = \left[\frac{S}{a_2^*}\right]$, we know that $S < (k_2+1)a_2^*$ and hence $F_A \leq S\psi_1(a_1^*) < (k+1)a_2^*\psi_1(a_1^*)$.

Consider another distribution of S, which is characterized by $S_2 = k_2 a_2^*$ and $S_1 = S - k_2 a_2^*$. In this case, the total fitness F_B is expressed as $F_1(S_1) + F_2(S_2) = F_1(S_1) + n_2^* f_2\left(\frac{ka_2^*}{n_2^*}\right) = F_1(S_1) + k_2 f_2(a_2^*) \geq k_2 f_2(a_2^*)$ as $n_2^* = k_2$ where $S_2 = k_2 a_2^*$ by **Theorem 2**.

Therefore, $F_B - F_A \geq k_2 f_2(a_2^*) - (k_2+1)a_2^*\psi_1(a_1^*) = (k_2+1)a_2^*\left(\frac{k_2}{k_2+1}\psi_2(a_2^*) - \psi_1(a_1^*)\right)$ which is larger than zero because $k_2 \geq 1$ as $S \geq a_2^*$ and hence $\frac{k_2}{k_2+1}\psi_2(a_2^*) \geq \frac{1}{2}\psi_2(a_2^*) > \psi_1(a_1^*)$. This indicates $F_A \neq F^*(S)$, therefore, by reduction to absurdity, $S_1^* \neq S$. Hence, $S_2^* > 0$.

Lemma 5.2

If $a_1^* < a_2^*$, $\frac{f_1(a_1^*)}{a_1^*} < 2\frac{f_2(a_2^*)}{a_2^*}$, and $S > a_2^*$, then $S_1^* < a_2^*$ given that $N_2 \geq 2(k+1)$ where $ka_2^* \leq S < (k+1)a_2^*$.

Proof

The total fitness $F^*(S)$ satisfies $F^*(S) = F_1(S_1^*) + F_2(S_2^*) = n_1^* f_1\left(\frac{S_1^*}{n_1^*}\right) + F_2(S_2^*) = \psi_1\left(\frac{S_1^*}{n_1^*}\right)S_1^* + F_2(S_2^*) \leq \psi_1(a_1^*)S_1^* + F_2(S_2^*)$.

Suppose that there is $F_A = F_1(S_1^*) + F_2(S_2^*)$ with $S_1^* \geq a_2^*$. Let us consider another distribution of S, which is characterized by $S_1 = S_1^* - ma_2^*$ where $m = [S_1^*/a_2^*]$. In this case, ma_2^* is equitably distributed to m offspring in the group of *Sex 2* which is not cared of in the aforementioned distribution (S_1^*, S_2^*). The total fitness

in this case is $F_B = F_1(S_1^* - ma_2^*) + mf_2(a_2^*) + F_2(S_2^*) \geq mf_2(a_2^*) + F_2(S_2^*)$.

Since $S_1^* < (m+1)a_2^*$ and $F_1(S_1^*) \leq \psi_1(a_1^*)S_1^*$, it holds that $F^*(S) \leq \psi_1(a_1^*)S_1^* + F_2(S_2^*) < \psi_1(a_1^*)(m+1)a_2^* + F_2(S_2^*)$. Here, note that $m \geq 1$ as $S_1^* \geq a_2^*$, which implies that $\psi_1(a_1^*)(m+1)a_2^* \leq mf_2(a_2^*)$ because $2\psi_1(a_1^*) < \psi_2(a_2^*)$. Therefore, $F_A < \psi_1(a_1^*)(m+1)a_2^* + F_2(S_2^*) \leq mf_2(a_2^*) + F_2(S_2^*) \leq F_B$, indicating that $F_A < F_B$. Thus, F_A is not optimal and by reduction to absurdity, $S_1^* < a_2^*$.

Definition 5

For a pair of logistic fitness functions, define $R_1 = \{f_1'(x) \mid 0 \leq x \leq 2p_1^*\}$. Define $I_1 = \{x \mid f_2'(x) \in R_1, x \leq p_2^*\}$ and $I_2 = \{x \mid f_2'(x) \in R_1, x \geq p_2^*\}$. Also, define $q_1 = \max(f_1''(x))$ for $0 \leq x \leq 2p_1^*$.

Theorem 5 (Strict condition for reversed TWH with respect to the investment)

Assume that the following conditions hold.

(1) $a_1^* < a_2^*$, $2\frac{f_1(a_1^*)}{a_1^*} < \frac{f_2(a_2^*)}{a_2^*}$, $f_1'(p_1^*) < f_2'(p_2^*)$ and $S > a_2^*$.

(2) $N_1 > S/a_1^*$ and $N_2 \geq 2(k+1)$ where $ka_2^* \leq S < (k+1)a_2^*$.

(3) There exists w_2 $(a_2^* < w_2 < min(I_2))$ such that $|f_2''(x)| > q_1$ for $x \in [w_2, \min(I_2)] \cup I_2$.

Then $S_2^* > S_1^*$ holds.

Proof

Due to condition (1) and (2), by **Lemma 5.1**, $S_2^* > 0$. If $S_1^* = 0$, then the theorem trivially holds. If $S_1^* > 0$, then by **Lemma 1**, $f'_1(v_1) = f'_2(v_2)$ where v_1 and v_2 are the non-zero optimal solutions. For brevity, as in **Definition 3**, denote by L_k and H_k the values which satisfy $\frac{S_k}{k+1}$ and $\frac{S_k}{k}$, respectively, such that $(k+1)\ f_1\left(\frac{S_k}{k+1}\right) = k\ f_1\left(\frac{S_k}{k}\right)$ holds. By **Lemma 2** and **Lemma 3**, $v_1 = \frac{S_1^*}{n_1^*} < H_1 = 2p_1^*$.

Take w_1 as the value satisfying $\frac{w_1+w_2}{2} = p_2^*$. For brevity, denote by $I_E = I_1 \cup [\max(I_1), w_1]$.

Suppose that $v_2 \in I_1$, then total fitness of this condition is $F_A = n_1^* f_1\left(\frac{S_1^*}{n_1^*}\right) + n_2^* f_2\left(\frac{S_2^*}{n_2^*}\right)$. Take $h > 0$ such that $\frac{S_2^*}{n_2^*} + h \in I_E$. Then, $f_2(v_2+h) = f_2(v_2) + \int_{v_2}^{v_2+h} f_2'(u)du$ where $f_2'(u) = f_2'(v_2) + \int_{v_2}^{u} f_2''(w)dw$. Therefore,

$$f_2(v_2+h) = f_2(v_2) + \int_{v_2}^{v_2+h}\left[f_2'(v_2) + \int_{v_2}^{u} f_2''(w)dw\right]du = f_2(v_2) + f_2'(v_2)h + \int_{v_2}^{v_2+h}\int_{v_2}^{u} f_2''(z)dz\,du$$

$$> f_2(v_2) + f_2'(v_2)h + \int_{v_2}^{v_2+h}\int_{v_2}^{u} q_1 dz\,du = f_2(v_2) + f_2'(v_2)h + q_1 h^2$$

because $\int_{v_2}^{v_2+h}\int_{v_2}^{u} q_1 dz\,du = q_1\int_{v_2}^{v_2+h}(u - v_2)du = q_1\left(\frac{(v_2+h)^2 - v_2^2}{2} - v_2 h\right) = q_1 h^2$.

Similarly,

$$f_1(v_1 - h) = f_1(v_1) - f_1'(v_1)h + \int_{v_1}^{v_1-h}\int_{v_1}^{u} f_1''(z)\,dz\,du$$

$$< f_1(v_1) - f_1'(v_1)h + \int_{v_1}^{v_1-h}\int_{v_1}^{u} q_1\,dz\,du = f_1(v_1) - f_1'(v_1)h - q_1h^2.$$

Hence,

$$f_2(v_2+h) - f_2(v_2) > f_2'(v_2)h + q_1h^2 = f_1'(v_1)h + q_1h^2 > f_1(v_1) - f_1(v_1-h)$$

and

$$f_2(v_2+h) + f_1(v_1-h) - f_2(v_2) - f_1(v_1) > 0$$

indicating that v_1 and v_2 are not optimal. By reduction to absurdity, $v_2 \notin I_1$.

As $S_2^* > 0$ while $f_1'(v_1) = f_2'(v_2)$ should hold, $v_2 \in I_2$. Therefore, $v_2 > a_2^*$ and $S_2^* \geq v_2 > a_2^*$. As $n_2^* \leq \left\lfloor \frac{S}{a_2^*} \right\rfloor + 1 = k + 1$, by the assumption, $N_2 - n_2^* \geq k$. By **Lemma 5.2**, there is a contradiction if $S_1^* \geq a_2^*$, therefore, $S_1^* < a_2^*$. As it was shown that $S_2^* \geq v_2 > a_2^*$, it should hold that $S_1^* < a_2^* < S_2^*$.

Lemma 6

If $a_1^* < a_2^*$, $S \leq \min(\{2p_1^*, p_2^*\})$, and $f_1'(0) > f_2'(S)$, then $S_1^* = S$ and $S_2^* = 0$ although $\psi_1(a_1^*) < \psi_2(a_2^*)$.

Proof

Note that $f_1'(x) \geq f_1'(0)$ for any $x \in [0, 2p_1^*]$ due to the symmetry of the fitness function, and $f_2'(x) \leq f_2'(S)$ for any $x \leq S$ because $S \leq p_2^*$.

If both n_1^* and n_2^* are non-zero, then **Lemma 1** implies there exist some v_1 and v_2 in $[0, S]$ such that $f_1'(v_1) = f_2'(v_2)$ holds. However, this cannot be true because $f_1'(v_1) \geq f_1'(0) > f_2'(S) \geq f_2'(v_2)$. Thus $n_1^* = 0$ or $n_2^* = 0$ must hold.

Suppose $n_1^* = 0$. Then the following should hold

$$\begin{aligned} F(S) = F_1^*(0) + F_2^*(S) = n_2^* f_2\left(\frac{S}{n_2^*}\right) &= n_2^* \int_0^{\frac{S}{n_2^*}} f_2'(u)du \\ &< n_2^* \int_0^{\frac{S}{n_2^*}} f_2'(S)du = Sf_2'(S) \\ &< Sf_1'(0) = \int_0^S f_1'(0)du \\ &< \int_0^S f_1'(u)du = f_1(S) \end{aligned}$$

which implies that $F(S)$ is not optimal and hence the assumption is false. That is, $n_1^* \neq 0$ and $n_2^* = 0$ and therefore $S_1^* = S$ and $S_2^* = 0$.

Remark 3

Although *Sex 2* has a higher maximal tangent through the origin, it is possible that the larger number of *Sex*

1 receive the care while the total proportion to *Sex-2* is greater.

Consider a pair of fitness functions defined by coefficients in logistics functions as:

Sex 1: $\alpha_1 = 10.8208, \beta_1 = 0.5, \gamma_1 = 5, \delta_1 = -0.8208;$

Sex 2: $\alpha_2 = 200, \beta_2 = 5, \gamma_2 = 5, \delta_2 = 0$

in $f_i(x) = \frac{\alpha_i}{1+e^{-\beta_i(x-\gamma_i)}} + \delta_i$.

Suppose that there are 5 *Sex 1* and 5 *Sex 2* offspring. The optimal number of cared offspring and optimal proportion of total care against *S* are shown in Fig. S34. For the certain domain of *S*, more number of *Sex 1* is cared of while the total investment (equivalent to the proportion) is greater for *Sex 2*.

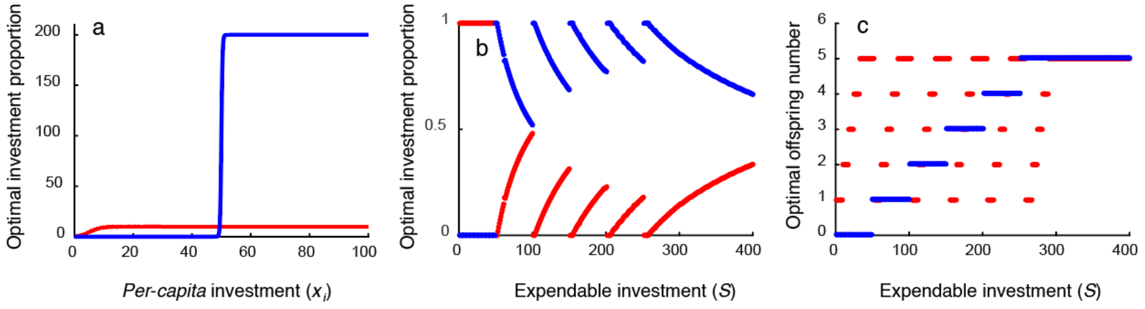

Figure S34. The example presented in Theorem 6. **(a)** A pair of fitness functions with coefficients described in Theorem 6. **(b)** Optimal proportion of investment of *Sex 1* and *Sex 2*. **(c)** The optimal number of cared offspring. There is a range of S where the total proportion is greater for *Sex 2*, but the cared number is greater for *Sex 1*.

Theorem 6 (Outright equitable distribution)

Suppose that there is L such that $1 < x < L$ indicates that $f_1(x) > f_2(x)$ where f_1 and f_2 are the fitness function of *Sex 1* and *Sex 2*, respectively. For that L, suppose that $x < L$ indicates that $f_1(x) < f_2(x)$. Suppose that investment is equitably distributed to all offspring of size N. Then total fitness of the *Sex-1*-biased brood is greater than that of the *Sex-2*-biased brood if $N < S < NL$. The opposite is true if $S > NL$.

Proof

Suppose that there are N offspring in 2 groups, named group 1 and group 2. For group 1, there are N_1 *Sex 1* offspring and N_2 *Sex 2* offspring. For group 2, there are M_1 and M_2 offspring of *Sex 1* and *Sex 2*, respectively.

Let group 1 be Sex-1-biased and group 2 be Sex-2-biased. That is,

$$N = N_1 + N_2 = M_1 + M_2$$

and

$$N_1 > M_1,\ N_2 < M_2.$$

Take $D = N_1 - M_1 = M_2 - N_1$. Denote F_A, F_B by the fitness of *Sex-1*-biased and *Sex-2*-biased brood, respectively. Since we assume that investment is equitably distributed, the investment each offspring receives is

$\frac{S}{N}$. Let us denote $u = \frac{S}{N}$. Then $F_A - F_B$ is expressed as following

$$F_A - F_B = \left(N_1 f_1(u) + N_2 f_2(u)\right) - \left(M_1 f_1(u) + M_2 f_2(u)\right) = D f_1(u) - D f_2(u) = D\left(f_1(u) - f_2(u)\right).$$

If $1 < u < L$, then $D\left(f_1(u) - f_2(u)\right) = F_A - F_B > 0$ indicating that sex-1-biased brood has higher fitness. In this condition, $N < S = Nu < NL$. On the other hand, if $u > L$, then $D\left(f_1(u) - f_2(u)\right) = F_A - F_B < 0$ so that *Sex-2*-biased brood has higher fitness. In this condition, $S = Nu > NL$.

Table S5. Explanation of the principal notation used in the mathematical analyses.

Variable/function	Description
f_i	The fitness function of i-th group.
p^*	The inflection point of f.
X^*	The optimized distribution of parental investment.
v_i	The optimized amount of parental investment given to the i-th offspring.
β_i	Components in the X^* which are less than p^* (not including 0).
γ_i	Components in X^* which are greater than p^*.
S	Total amount of expendable parental investment.
$\psi(x)$	$f(x)/x$, the efficiency of parental care.
n^*	The optimized number of offspring who are receiving investment.
N	The total number of offspring.
k	$[S/a^*]$, the integer not bigger than S/a^*.
$\Psi(S)$	$\psi\left(\frac{S}{k+1}\right) - \psi\left(\frac{S}{k}\right)$
L_k	$\frac{S_k}{k+1}$ such that $(k+1)\ f\left(\frac{S_k}{k+1}\right) = k\ f\left(\frac{S_k}{k}\right)$ holds.
H_k	$\frac{S_k}{k}$ such that $(k+1)\ f\left(\frac{S_k}{k+1}\right) = k\ f\left(\frac{S_k}{k}\right)$ holds.
$F_i(X)$	Optimal offspring fitness of i-th group to which X amount of care is given.
$F(S_1, S_2)$	$F_1(S_1) + F_2(S_2)$, the total optimal offspring fitness.
$\phi(K)$	α/β where α and β are solution of $\psi(x) = K$
R_1	$\{f_1'(x)\ \mid\ 0 \le x \le 2p_1^*\}$
I_1	$\{x\ \mid\ f_2'(x) \in R_1, x < p_2^*\}$
I_2	$\{x\ \mid\ f_2'(x) \in R_1, x > p_2^*\}$
q_1	$\max_{x \in [0, 2p_1^*]} f_1''(x)$

References for Supplementary Materials

1. Borgstede, M. (2019). Is there a Trivers–Willard effect for parental investment? Modelling evolutionarily stable strategies using a matrix population model with nonlinear mating. *Theoretical population biology*, *130*, 74-82.
2. Cameron, E. Z., & Linklater, W. L. (2002). Sex bias in studies of sex bias: the value of daughters to mothers in poor condition. *Animal behaviour*, *63*(2), F5.
3. Carranza, J. (2002). What did Trivers and Willard really predict?. *Animal Behaviour*, *63*(2), F1-F3.
4. Cockburn, A., Legge, S., & Double, M. C. (2002). Sex ratios in birds and mammals: can the hypotheses be disentangled. *Sex ratios: concepts and research methods*, 266-286.
5. Krist, M. (2006). Should mothers in poor condition invest more in daughter than in son?. *Ethology Ecology & Evolution*, *18*(3), 241-246.
6. Leimar, O. (1996). Life-history analysis of the Trivers and Willard sex-ratio problem. *Behavioral Ecology*, *7*(3), 316-325.
7. Pen, I., & Weissing, F. J. (2002). Optimal sex allocation: steps towards a mechanistic theory. *Sex ratios: concepts and research methods. Cambridge University Press, Cambridge*, 26-45.
8. Rosenheim, J. A., Nonacs, P., & Mangel, M. (1996). Sex ratios and multifaceted parental investment. *The American Naturalist*, *148*(3), 501-535.
9. Schindler, S., Gaillard, J. M., Grüning, A., Neuhaus, P., Traill, L. W., Tuljapurkar, S., & Coulson, T. (2015). Sex-specific demography and generalization of the Trivers–Willard theory. *Nature*, *526*(7572), 249-252.
10. Shyu, E., & Caswell, H. (2018). Mating, births, and transitions: a flexible two-sex matrix model for evolutionary demography. *Population ecology*, *60*(1), 21-36.
11. Tinbergen, N. (1963). On aims and methods of ethology. *Zeitschrift für tierpsychologie*, *20*(4), 410-433.
12. Veller, C., Haig, D., & Nowak, M. A. (2016). The Trivers–Willard hypothesis: sex ratio or investment?. *Proceedings of the Royal Society B: Biological Sciences*, *283*(1830), 20160126.
13. Wild, G., & West, S. A. (2007). A sex allocation theory for vertebrates: combining local resource competition and condition-dependent allocation. *The American Naturalist*, *170*(5), E112-E128.
