# Supplementary material for: Reformulation of Trivers–Willard hypothesis for parental investment
Source: Commun Biol. 2022 Apr 19;5:371. doi: 10.1038/s42003-022-03286-z (PMC9018816; doi:10.1038/s42003-022-03286-z)
Supplement: Supplementary file 3 — Description of Additional Supplementary Files [file 42003_2022_3286_MOESM3_ESM.pdf]

Description of Additional Supplementary Files

File name: Supplementary Animation S1.
Description: The relationship between the slope of the tangent lines through the origin (left) and optimal investment proportion (right).

The sex that has a steeper slope is preferred (more proportion of parental investment to the whole offspring of that sex).

File name: Supplementary Animation S2.
Description: Same as Supplementary Animation S1 with a different configuration.
