# Supplementary figures and images for: Reformulation of Trivers–Willard hypothesis for parental investment
Source: Commun Biol. 2022 Apr 19;5:371. doi: 10.1038/s42003-022-03286-z (PMC9018816; doi:10.1038/s42003-022-03286-z)

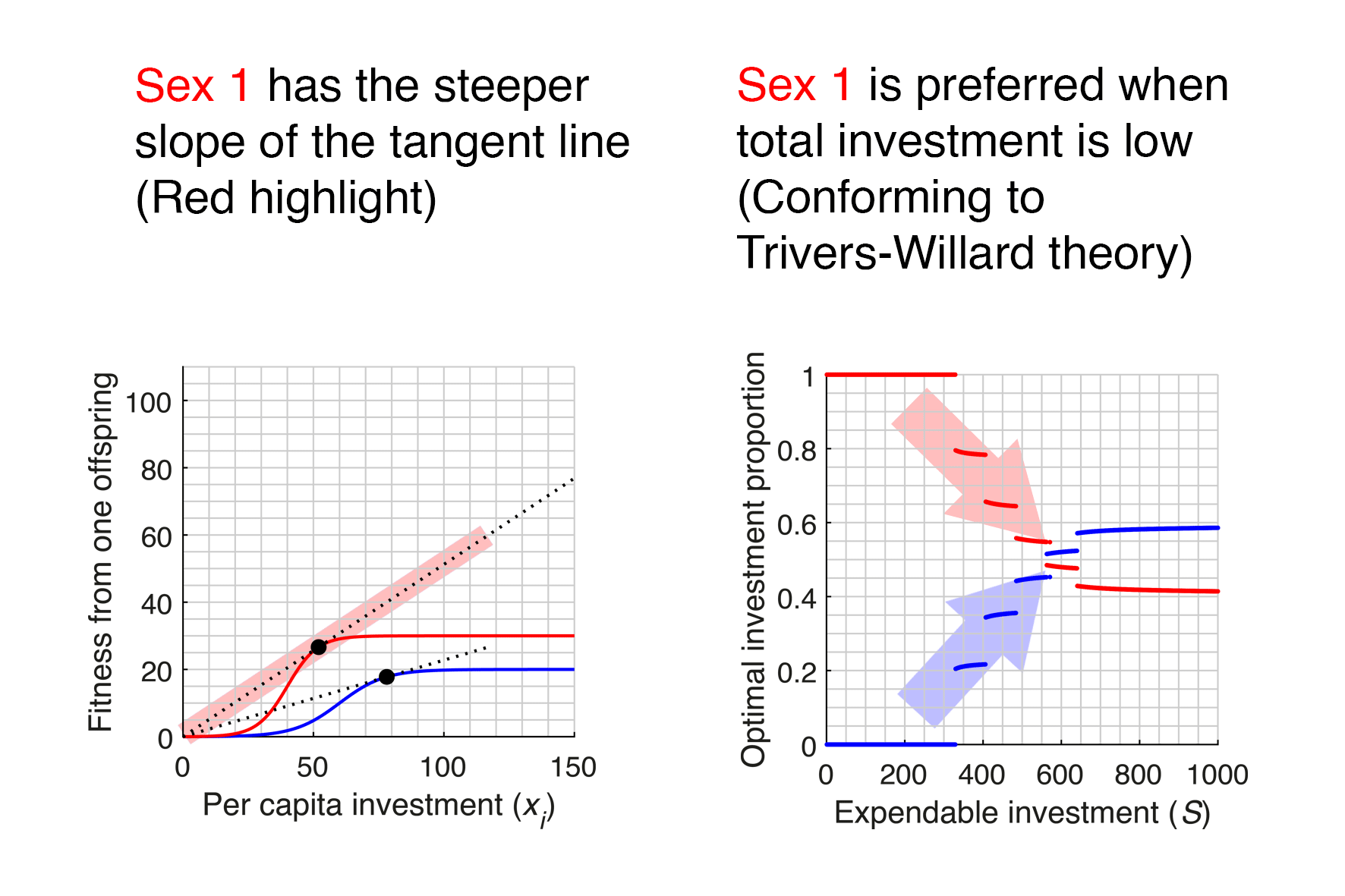

Supplement: Supplementary file 4 — Supplementary Animation S1 [file 42003_2022_3286_MOESM4_ESM.gif]

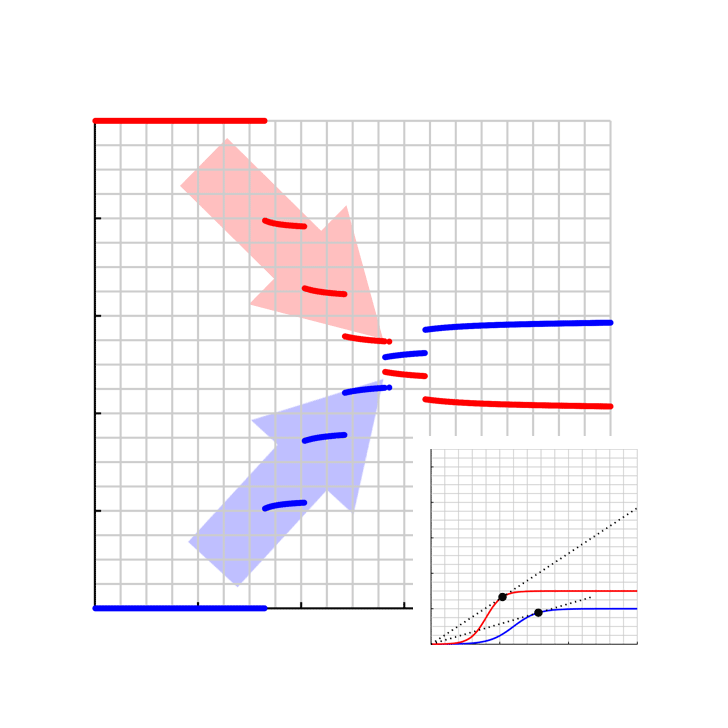

Supplement: Supplementary file 5 — Supplementary Animation S2 [file 42003_2022_3286_MOESM5_ESM.gif]
